# PMK-1 p38 MAPK promotes cadmium stress resistance, the expression of SKN-1/Nrf and DAF-16 target genes, and protein biosynthesis in *Caenorhabditis elegans*

**DOI:** 10.1007/s00438-017-1351-z

**Published:** 2017-08-01

**Authors:** Alex Keshet, Ansgar Mertenskötter, Sarah A. Winter, Vanessa Brinkmann, Ramona Dölling, Rüdiger J. Paul

**Affiliations:** Institute of Zoophysiology, University of Münster (WWU), Schlossplatz 8, 48143 Münster, Germany

**Keywords:** Glutathione, Metallothionein, Molecular chaperones, RNA-Seq, TOR signaling, Transcriptome

## Abstract

The mechanisms of cadmium (Cd) resistance are complex and not sufficiently understood. The present study, therefore, aimed at assessing the roles of important components of stress-signaling pathways and of ABC transporters under severe Cd stress in *Caenorhabditis elegans*. Survival assays on mutant and control animals revealed a significant promotion of Cd resistance by the PMK-1 p38 MAP kinase, the transcription factor DAF-16/FoxO, and the ABC transporter MRP-1. Transcriptome profiling by RNA-Seq on wild type and a *pmk*-*1* mutant under control and Cd stress conditions revealed, inter alia, a PMK-1-dependent promotion of gene expression for the translational machinery. PMK-1 also promoted the expression of target genes of the transcription factors SKN-1/Nrf and DAF-16 in Cd-stressed animals, which included genes for molecular chaperones or immune proteins. Gene expression studies by qRT-PCR confirmed the positive effects of PMK-1 on DAF-16 activity under Cd stress and revealed negative effects of DAF-16 on the expression of genes for MRP-1 and DAF-15/raptor. Additional studies on *pmk*-*1* RNAi-treated wild type and mutant strains provided further information on the effects of PMK-1 on SKN-1 and DAF-16, which resulted in a model of these relationships. The results of this study demonstrate a central role of PMK-1 for the processing of cellular responses to abiotic and biotic stressors, with the promoting effects of PMK-1 on Cd resistance mostly mediated by the transcription factors SKN-1 and DAF-16.

**Electronic supplementary material:**

The online version of this article (doi:10.1007/s00438-017-1351-z) contains supplementary material, which is available to authorized users.

## Introduction

The heavy metal cadmium (Cd) poses a serious threat to organisms and cells by promoting the formation of reactive oxygen species (ROS; Wang et al. 2004) and impairing antioxidant systems, DNA repair, RNA/protein synthesis, cell cycle, differentiation, and proliferation (Beyersmann and Hechtenberg 1997; Company et al. 2004). As the natural habitat of the nematode *Caenorhabditis elegans* (i.e., pore water of humus soil) can be contaminated with Cd and other toxins in varying concentrations, the worm had to develop stress-protective mechanisms against these toxic substances and other stressors during its evolution (Martinez-Finley and Aschner 2011). Stress response mechanisms are a universal tool for survival in harmful conditions. The manifold (experimental) advantages of the model organism *C. elegans*, including a high degree of genetic similarity with higher organisms (including humans), create the possibility to study principles of stress resistance in a comparatively simple organism.

Stress responses require, first and foremost, signal processing, with mitogen-activated protein kinase (MAPK) and insulin-like (DAF-2) signaling being particularly important for the processing of stress and other signals in animal cells. The JNK-like MAPK KGB-1 of *C. elegans* participates in the response to heavy metal stress (Koga et al. 2000; Mizuno et al. 2008; Fujiki et al. 2010), which involves phosphorylation of the transcription factor FOS-1 and the regulation of two so far known genes (*kreg*-*1,* -*2*; Hattori et al. 2013). For the p38-type MAPK PMK-1, a participation in Cd-induced germline apoptosis has also been shown (Wang et al. 2008). At present, however, PMK-1 is especially known for its participation in pathogen resistance mechanisms (Huffman et al. 2004; Troemel et al. 2006; Bolz et al. 2010) and oxidative or heat stress responses (An et al. 2005; Mertenskötter et al. 2012). PMK-1-dependent signal processing includes (1) activation of the Nrf-like transcription factor SKN-1 (Inoue et al. 2005), which is a major regulator of genes for detoxification and antioxidant defense, (2) PMK-1 nuclear translocation (Mertenskötter et al. 2012), and (3) deactivation of the transcriptional repressor ATF-7 in context with pathogen resistance mechanisms (Shivers et al. 2010).

Reduced DAF-2 signaling, resulting in nuclear translocation of the transcription factor DAF-16/FoxO, also contributes to heavy metal resistance by promoting, for instance, gene expression for heat shock proteins, metallothioneins, and antioxidant enzymes (Barsyte et al. 2001). Furthermore, the transcription factors SKN-1 and DAF-16 have been identified as central nodes of general stress resistance and even longevity (Tullet et al. 2008; Kenyon 2010; Robida-Stubbs et al. 2012). ABC transporters also significantly contribute to heavy metal resistance. These include the multidrug resistance-associated protein 1 (MRP-1) and P-glycoprotein 1 (PGP-1) (Broeks et al. 1996) as well as the half-molecule ABC transporter HMT-1 (heavy metal tolerance factor 1), which promotes the sequestration of heavy metal-binding phytochelatins (PCs) (Vatamaniuk et al. 2001, 2005). The Cd-responsive protein CDR-1 further improves Cd resistance, possibly by pumping Cd^2+^ into lysosomes (Liao et al. 2002). Survival experiments on wild type and mutant strains under Cd stress have revealed several other genes contributing to Cd resistance (Roh et al. 2009), and diverse genes, in turn, have shown Cd-induced expression (Roh et al. 2006). Transcriptome profiling by microarray analyses revealed several hundred Cd-inducible genes, including *cdr*-*1* and *mtl*-*1/2* (metallothioneins) as well as *cyp* (cytochrome P450) and *gst* (glutathione *S*-transferase) genes (Cui et al. 2007). A recent study, however, emphasized the importance of HMT-1 for the Cd resistance of *C. elegans* in comparison to the importance of CDR- or MTL-based mechanisms (Hall et al. 2012).

The present study was carried out to analyze the role of PMK-1 signaling in the Cd stress responses of *C. elegans*, while also focusing on interactions with DAF-16 activity and functions of ABC transporters (MRP-1, PGP-1). To activate the full spectrum of Cd stress responses, severe Cd stress was applied. After determining the survival rates of WT, stress-signaling or ABC transporter mutants, *pmk*-*1* RNAi-treated worms, and strains with overexpressed genes/proteins for PMK-1- and DAF-16-dependent gene control, RNA-Seq (next-generation sequencing) and bioinformatic tools were applied to identify differentially expressed genes (DEGs) in WT and *pmk*-*1*Δ under control conditions and severe but not lethal Cd stress. The decrease in DAF-16 target gene expression in *pmk*-*1*Δ under Cd stress required additional experiments on gene expression and DAF-16 subcellular localization. The present study provides evidence for multiple and interacting ways of protection against severe Cd stress, strongly depending on PMK-1 signaling and DAF-16 target gene expression, and revealed promoting effects of PMK-1 on protein biosynthesis.

## Materials and methods

### Wild type, mutant, and transgenic strains

The N2 Bristol variety of *C. elegans* (wild type, WT) and different mutants, hereinafter referred to as *pmk*-*1*Δ [KU25, *pmk*-*1(km25)* IV], *daf*-*2*Δ [CB1370, *daf*-*2(e1370)* III], *daf*-*16*Δ [CF1038, *daf*-*16(mu86)* I], *mrp*-*1*Δ [NL147, *mrp*-*1(pk89)*], *mrp*-*2*Δ [RB1713, *mrp*-*2(ok2157)* X], *pgp*-*2*∆ [VC134, *pgp*-*2(gk114)* I], *pgp*-*1*/*pgp*-*3*Δ [NL130, *pgp*-*1(pk17)* IV; *pgp*-*3(pk18)* X], and *mrp*-*1*/*pgp*-*1*/*pgp*-*3*Δ [NL152, *pgp*-*1(pk17)* IV; *pgp*-*3(pk18)* X; *mrp*-*1(pk89)*] were obtained from the Caenorhabditis Genetics Center (CGC; http://www.cbs.umn.edu/CGC/). The TJ356 (*zIs356* IV) reporter strain, which carries a genome-integrated *daf*-*16*::*gfp* construct, was also obtained from the Caenorhabditis Genetics Center. Generation of the reporter strain PMK-1::GFP, which carries a *pmk*-*1*::*gfp* construct, has been described elsewhere (Mertenskötter et al. 2012). Worm cultures were maintained at 20 °C on NGM plates with *E. coli* OP50 as the food source.

### RNA interference

For gene knockdown, RNAi was performed by feeding dsRNA-expressing *E. coli* HT115 strains to *C. elegans*. Two *E. coli* HT115 strains were provided by Source BioScience LifeSciences (Nottingham, UK): *E. coli* HT115, *dpy*-*7* [F46C8.6] RNAi (phenotypic control of IPTG impact), and *E. coli* HT115, control (empty vector L4440) RNAi. Generation of the *E. coli* HT115, *pmk*-*1* [B0218.3] RNAi strain was described in Mertenskötter et al. (2012). Bacteria were grown overnight at 37 °C in LB medium plus ampicillin (100 µg/mL). NGM plates, containing 1 mmol/L isopropyl-β-d-1-thiogalactopyranoside (IPTG), were seeded with 1 mL of bacterial suspension (different *E. coli* HT115 strains; OD_600nm_ = 1) and incubated overnight at 20 °C to induce dsRNA expression of the bacteria. Then, synchronized L1 larvae were transferred to these plates for growing. Gene knockdowns by *pmk*-*1* RNAi treatment were verified by quantitative RT-qPCR (see below and Table 1). The ratios between the mRNA levels of *pmk*-*1* and *cdc*-*42* (housekeeping gene) were clearly below 1% in all *pmk*-*1* RNAi-treated worms in comparison to the respective control worms, which were fed with *E. coli* HT115, control (empty vector L4440).

**Table 1:** RT-sqPCR and RT-qPCR amplification conditions and primer pairs for (top) RT-sqPCR of selected mRNA species, (center) RT-sqPCR of standards for RT-qPCR, and (bottom) RT-qPCR of selected mRNA species

Gene symbol	Forward primer	Reverse primer	Length (bp)	Denaturation at 94 °C (s)	Annealing time (s)	*T*_a_ (°C)	Elongation at 72 °C (s)	Cycles
*cdc*-*42*	ATGCAGACGATCAAGTGCG	TTCAGTCCCTTCTGCGTCA	497	30	45	53	35	28
*daf*-*15*	TATAGAGAACGAG	GTGAACGAACTA	525	30	45	61	45	31
*mrp*-*1*	CTCCTGTCCAGAATACAC	TCCCATAGGACGTAGAG	1021	30	45	53.7	60	29
*mtl*-*1*	ATGGCTTGCAAGTGTG	TTAATGAGCCGCAGC	228	30	45	57.9	20	33
*mtl*-*2*	ATGGTCTGCAAGTGTG	TTAATGAGCAGCCTGA	192	30	45	57	20	30
*ttm*-*1*	TAGCAATTATGACAGACGCC	AAACGCAGTCTTATCCATTCC	680	45	30	57.9	40	36

Gene symbol	Forward primer	Reverse primer	Length (bp)	Denaturation at 94 °C (s)	Annealing time (s)	*T*_a_ (°C)	Elongation at 72 °C (s)	Cycles
*cdc*-*42*	TTCGACAATTACGCCGTCAC	CCTGAGATCGACTTGAGTACC	251	20	40	59	40	31
*ctl*-*2*	CCATTTCAAGCCAACTCAAGGA	ACATCTTCCATACTGGAAAGTCTC	136	20	40	59	40	31
*dhs*-*18*	CGAAATTGAGAAGGCAGGAGG	CATCAGATCGTATCGTTTCATGTC	186	20	40	59	40	31
*hsp*-*12.6*	GAGTTGTCAATGTCCTCGAC	ATTCCATGTGAATCCAAGTTGC	234	20	40	59	40	31
*hsp*-*17*	ATGGACAGGTAGAGCGTCAC	GCTGCTGTTCCTGATTCTTCTC	131	20	40	59	40	31
*nit*-*1*	TTGCATTAGAAGGAAGATGCTTCG	GACCTTCAACTGGAATACATCCG	283	20	40	59	40	31
*pmk*-*1*	ATCATATACTTCATCCGACTCCAC	TACATTCAGCAGCACAAACAG	140	20	40	59	40	31
*skr*-*5*	GTAGAGTTCATTGTTTCTCCGA	ATCTTCAGCATGATACTCACAC	152	20	40	59	40	31
*ugt*-*1*	GGAGCTATAGCAACTTCAACAG	TGTTAAGCTTCAAGTGATCTCC	171	20	40	59	40	31

Gene symbol	Forward primer	Reverse primer	Length (bp)	Denaturation at 95 °C (s)	Annealing time (s)	*T*_a_ (°C)	Cycles
*cdc*-*42*	TTCGACAATTACGCCGTCAC	CCTGAGATCGACTTGAGTACC	251	10	30	59	40
*ctl*-*2*	CCATTTCAAGCCAACTCAAGGA	ACATCTTCCATACTGGAAAGTCTC	136	10	30	59	40
*dhs*-*18*	CGAAATTGAGAAGGCAGGAGG	CATCAGATCGTATCGTTTCATGTC	186	10	30	59	40
*hsp*-*12.6*	GAGTTGTCAATGTCCTCGAC	ATTCCATGTGAATCCAAGTTGC	234	10	30	59	40
*hsp*-*17*	ATGGACAGGTAGAGCGTCAC	GCTGCTGTTCCTGATTCTTCTC	131	10	30	59	40
*nit*-*1*	TTGCATTAGAAGGAAGATGCTTCG	GACCTTCAACTGGAATACATCCG	283	10	30	59	40
*pmk*-*1*	ATCATATACTTCATCCGACTCCAC	TACATTCAGCAGCACAAACAG	140	10	30	59	40
*skr*-*5*	GTAGAGTTCATTGTTTCTCCGA	ATCTTCAGCATGATACTCACAC	152	10	30	59	40
*ugt*-*1*	GGAGCTATAGCAACTTCAACAG	TGTTAAGCTTCAAGTGATCTCC	171	10	30	59	40

Amplifications were always preceded by a pre-heating step (95 °C, 5 min). Primers were designed using the PerlPrimer program (Marshall 2004)

*T*_a_ annealing temperature

### Survival assays

Synchronized young adult worms were transferred to control (without CdCl_2_) and/or test (addition of 10 mmol/L CdCl_2_) plates (NGM; *T* = 20 °C) seeded with either *E. coli* OP50 (WT and mutants) or *E. coli* HT115 (control and *pmk*-*1* RNAi treatment of WT and mutants), and survival was tested after different incubation periods (1–24 h) by applying gentle touch stimuli with a worm pick, with worms not responding scored as dead.

### RNA-Seq

Many hundred synchronized young adult WT and *pmk*-*1*Δ worms were incubated for 5 h on control (without CdCl_2_) or test (10 mmol/L CdCl_2_) plates (∅ = 90 mm; NGM; *T* = 20 °C) seeded with *E. coli* OP50 (per condition and strain, two plates with animals from independent experiments). The following procedure has already previously been described (Mertenskötter et al. 2012). Briefly, worms were washed from the plates after incubation and cleaned with purified water. After adding RNAiso-G (Segenetic, Borken, Germany), they were frozen in liquid nitrogen. After multiple cycles of thermal disruption of worms (liquid nitrogen, 35 °C), chloroform extraction on ice, and centrifugation, the RNA was isolated and purified with an RNase-free DNase set and the RNeasy^®^ mini kit (Qiagen, Hilden, Germany). Quality control was carried out with an Agilent Bioanalyzer^®^ (Agilent Technologies, Böblingen, Germany). After adding the RNAstable™ matrix (Biomatrica, San Diego, CA, USA) and a subsequent vacuum centrifugation for drying, samples were sent to the Beijing Genomics Institute (BGI) for RNA-Seq analysis (see Mertenskötter et al. 2012 for details). Using Illumina HiSeq 2000 technology, samples were sequenced with a minimum of 10 megareads per sample and a sequencing quality of more than 98% clean reads. Sequences were mapped to Wormbase release WS223. Differential gene expression was calculated using the RPKM method (reads per kilobase per million reads) out of the number of reads for one gene, the transcript length, and the overall number of reads in the sample (Mortazavi et al. 2008). *P* values of DEGs were determined referring to Audic and Claverie (1997), and the false discovery rate (FDR) was used to determine the threshold of *P* for the DEGs. We took an FDR <0.005 as the threshold for DEGs.

### Quantitative and semi-quantitative reverse transcription PCR (RT-PCR)

Synchronized young adult worms were washed from control plates using M9 buffer (Stiernagle, 2006), with some of them used for the determination of starting values for the time-resolved measurements (*daf*-*15* and *mrp*-*1* mRNA levels). The other worms were transferred to control (without CdCl_2_) and/or test (10 mmol/L CdCl_2_) plates (NGM; *T* = 20 °C), which were seeded with either *E. coli* OP50 (WT and mutants) or *E. coli* HT115 (control and/or *pmk*-*1* RNAi treatment of WT and mutants). After incubation between 1 and 14 h, the worms were washed from these plates and cleaned several times with purified water to exclude bacteria. Animals were instantly frozen with liquid nitrogen, and tissues and cells were broken by multiple cycles of thermal disruption of the worms (liquid nitrogen, 35 °C), providing the raw material for RNA extraction. Due to the unequal availability of equipment, the mRNA level of some genes (*daf*-*15*, *mrp*-*1*, *mtl*-*1*, *mtl*-*2*, *ttm*-*1*) was determined by semi-quantitative RT-sqPCR, whereas that of others (*nit*-*1*, *ctl*-*2*, *dhs*-*18*, *hsp*-*17*, *ugt*-*1*, *skr*-*5*, *hsp*-*12.6*) was determined by quantitative RT-qPCR. Total RNA was isolated using RNAiso-G. In the case of RT-qPCR, the RNA was additionally cleaned using peqGOLD Total RNA Kit/S-Line columns (PEQLAB Biotechnologie, Erlangen, Germany). After reverse transcription of 1 µg total RNA per sample using oligo(dT)_18_-primers (First Strand cDNA Synthesis Kit; Fermentas, St. Leon-Rot, Germany) and RevertAid Reverse Transcriptase (Thermo Fisher Scientific, Darmstadt, Germany), the cDNA quantities were determined using a NanoDrop spectrophotometer (Thermo Fisher Scientific) and included in the final calculations.

In the case of RT-sqPCR, the 50 µL of reaction medium for cDNA amplification contained 0.5 μL of template cDNA, 1 μL of the specific primer pair (10 mmol/L each), 1 μL of dNTP-mix (dATP, dTTP, dGTP, dCTP; 10 mmol/L each), 0.5 μL of Taq DNA-polymerase (5 U/μL), 5 μL of 10× Taq polymerase buffer (all components from Segenetic), and 42 µL of purified water. The PCR conditions are summarized in Table 1 (top). Quantification and analysis of band intensities were made using ImageJ 1.44 software (http://imagej.nih.gov/ij/). Ratios between the expression levels of the genes of interest and the housekeeping gene (*cdc*-*42*; Hoogewijs et al. 2008) were calculated and are shown.

For RT-qPCR, gene-specific standards were prepared by RT-sqPCR (see Table 1, center, for PCR conditions). The quantities of obtained products were determined using a NanoDrop spectrophotometer. Then, concentration series with steps of tenfold dilution were prepared using purified water. RT-qPCR was performed using a Real-Time PCR System (Eco Real-Time PCR; Illumina) and the PerfeCTa SYBR^®^ Green SuperMix Kit (Quanta Biosciences, Beverly, MA, USA). Each sample contained 1 μL of template cDNA, 5 μL of SYBR Green SuperMix, 1 μL of the specific primer pair (10 mmol/L each), and 3 μL of purified water, resulting in an end volume of 10 μL. The PCR conditions are summarized in Table 1 (bottom). A melting curve was generated after the cycling steps. To calculate the amount of mRNA, the Eco Real-Time PCR System software was used. Ratios were calculated between the mRNA levels of the genes of interest and the housekeeping gene (*cdc*-*42*).

### DAF-16::GFP localization assay

TJ356 worms were fed during growth with *E. coli* HT115 (TJ356, control RNAi and TJ356, *pmk*-*1* RNAi). For an improved view on the cell nuclei of all tissues, worms without eggs were used for experiments (synchronized L3 larvae, which may have developed to L4 larvae during experiments). Plates with a thin NGM layer, seeded with *E. coli* HT115 and containing either 0 mmol/L (control condition) or 10 mmol/L (test condition) CdCl_2_, were kept overnight at 20 °C. Then, four ‘pads’ were cut out from the NGM layer using a drinking straw, placed on a gas permeable lumox^®^ dish (Sarstedt, Nümbrecht, Germany), and surrounded with palmitic acid (10 mg/mL; BioXtra, Sigma-Aldrich, Germany) to prevent an escape of worms (Miller and Roth 2009). After lining the rim of the dish with a wet tissue to avoid desiccation and placing one worm on each pad, the dish was sealed with parafilm. Under a fluorescence microscope (Zeiss Axiovert), the TJ356 worms on the pads were regularly screened for DAF-16::GFP subcellular localization (cytoplasmic or intermediate and nuclear localizations) for a maximum of 8.5 h.

### Databases

For KOG (euKaryotic clusters of Orthologous Groups) classification, WormMart (Wormbase release WS220bugFix) was used to assign KOG (COG) codes of functional categories (http://www.ncbi.nlm.nih.gov/COG/grace/fiew.cgi) to the DEGs. David 6.8 (https://david-d.ncifcrf.gov/) was used for Gene-GO (gene ontology) analysis (functional annotation clustering), with the ease score (i.e., a modified Fisher exact *P* value) measuring gene enrichment in annotation terms.

### Statistics and computations

The data are given as the mean ± standard deviation (sd) or means of mean ± standard error (se) with *n* indicating the number of test groups (biological replicates). A one-way or two-way analysis of variance (anova) including a subsequent multiple comparison procedure (Holm–Sidak’s method) was used to test for differences in survival rate or mRNA expression level. In case of non-normally distributed data, Mann–Whitney rank-sum tests were used (i.e., tests for differences in absolute expression changes). Using Microsoft Excel 2007, Chi-square tests were applied to identify KOG categories with significant deviation (*P* < 0.05) in the ratio between the numbers of up- and downregulated DEGs from the ratio between the numbers of all up- and downregulated KOG-identified DEGs of a specific contrast (gene enrichment analysis). SigmaPlot 11.0 (Systat Software, Erkrath, Germany) was used for graph preparations and other statistical analyses.

## Results

### Survival rates of WT, mutants, gene-overexpressing strains, and *pmk*-*1* RNAi-treated worms under Cd stress

**Fig. 1:**
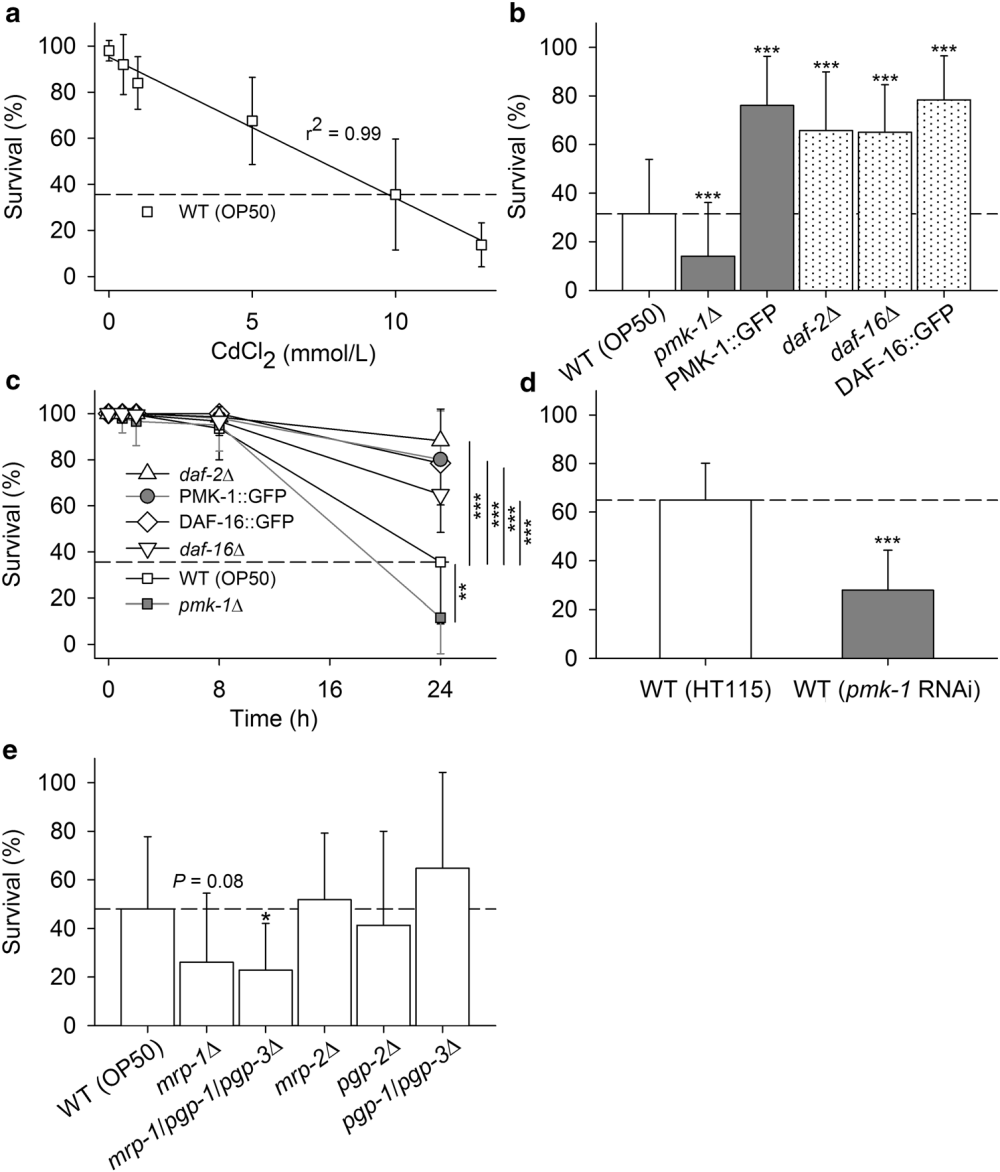
Survival rates of WT, stress-signaling or ABC transporter mutants, gene-overexpressing strains, and *pmk*-*1* RNAi-treated WT under Cd stress. Using either (**a**–**c**, **e**) *E. coli* OP50 [for WT (OP50) and mutants] or (**d**) *E. coli* HT115 [for control RNAi-(HT115) and *pmk*-*1* RNAi-treated WT] as the food source, the survival rates of the different *C. elegans* strains were determined under Cd stress. **a** The survival rate of WT (OP50) decreased linearly with increasing Cd concentration (24-h exposures at 0–13 mmol/L CdCl_2_; mean ± SD, per concentration, *n* = 3 test groups with 50 worms each; *diagonal line*, regression line; *dashed line*, survival rate at 10 mmol/L CdCl_2_). Testing the survival rates of (**b**) WT (OP50), stress-signaling mutants, and gene-overexpressing strains or (**d**) WT (HT115) and *pmk*-*1* RNAi-treated WT after 24 h at 10 mmol/L CdCl_2_ revealed Cd resistance to be lower in *pmk*-*1*∆ and WT (*pmk*-*1* RNAi) and higher in other mutant (*daf*-*2∆*, *daf*-*16∆*) or gene-overexpressing strains (PMK-1::GFP, DAF-16::GFP) than in the respective WT control [mean ± SD; per feeding condition (WT) and strain or treatment, *n* = 8–80 test groups with 10–15 worms each]. **c** Testing the survival rates of these strains at 10 mmol/L CdCl_2_ but now after different incubation periods resulted in similarly negative effects of Cd after 24 h; however, the survival rates remained near 100% at incubation periods below 8 h (mean ± SD; per strain and incubation period, *n* = 8–51 test groups with 10–15 worms each). **e** Testing the survival rates of WT (OP50) and ABC transporter mutants after 24 h at 10 mmol/L CdCl_2_ revealed a reduced Cd resistance of *mrp*-*1/pgp*-*1/pgp*-*3∆* and a trend (*P* = 0.08) towards a reduction in *mrp*-*1∆* in comparison to that in WT (OP50) (mean ± SD; per strain, *n* = 3–42 test groups with 15 worms each). For all tests (**b**–**e**), control experiments (without CdCl_2_) were carried out for all feeding conditions (WT), test animal groups, and incubation periods, which always resulted in survival rates close to 100% (data not shown). One-way anova and Holm–Sidak’s method were used for statistical analyses, and *asterisks* indicate significance levels (*P < 0.05; **P < 0.01; ***P < 0.001)

After determining the survival rates of synchronized young adult WT worms after 24 h at different Cd concentrations (Fig. 1a), 10 mmol/L CdCl_2_ was chosen as the standard test (survival rate approximately 36%), because it provided a sufficient margin to test for variations in stress resistance (higher or lower survival rates). Measuring the survival rates of stress-signaling mutants (*pmk*-*1*Δ, *daf*-*2*Δ, *daf*-*16*Δ), PMK-1- or DAF-16-overexpressing strains (PMK-1::GFP, DAF-16::GFP), and *pmk*-*1* RNAi-treated WT worms after 24 h under Cd stress (Fig. 1b, d) revealed negative effects of *pmk*-*1* knockout or knockdown and positive effects of *daf*-*2* or *daf*-*16* knockout and PMK-1 or DAF-16 overexpression on Cd resistance. Time-resolved experiments verified this result but additionally showed almost 100% survival at incubation periods below 8 h (Fig. 1c). Furthermore, the Cd resistance of WT worms (dashed lines) seemed to be affected by feeding conditions (Fig. 1a–c, *E. coli* OP50; Fig. 1d, *E. coli* HT115), which was verified by additional experiments (R. J. Paul; unpublished data). Testing survival rates of ABC transporter mutants after 24 h under Cd stress (Fig. 1e) revealed negative effects of a triple mutation (*mrp*-*1*/*pgp*-*1*/*pgp*-*3∆*) on Cd resistance. Because *pgp*-*1*/*pgp*-*3∆* survived Cd stress even slightly better than WT and because *mrp*-*1∆* showed a statistical trend towards lower Cd resistance, the negative effect of the triple mutation was most likely due to *mrp*-*1∆.*

### Transcriptomics

**Fig. 2:**
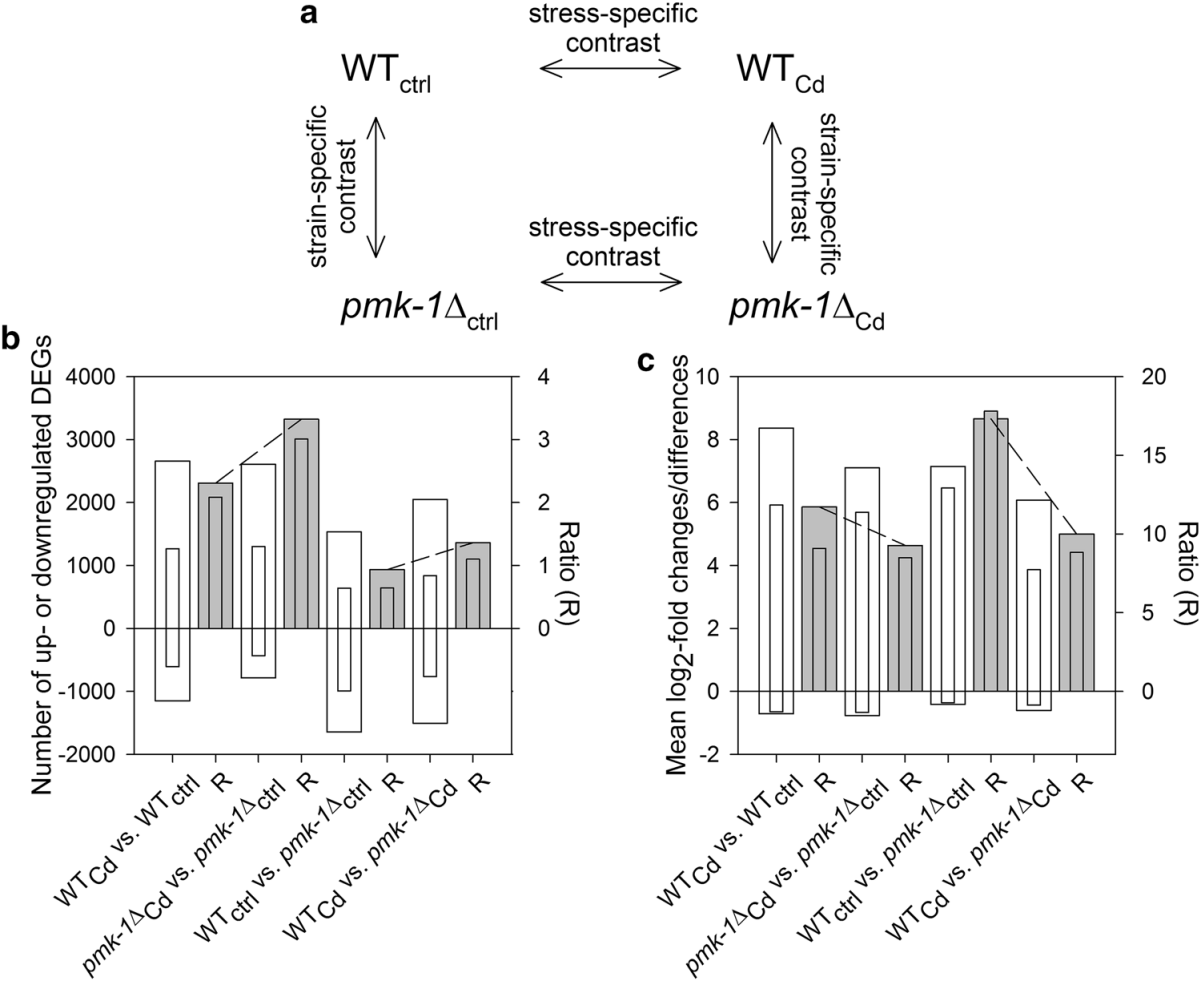
Numbers and mean log_2_-fold changes (or differences) for the expression of all DEGs and of KOG-identified DEGs in (or between) WT and *pmk*-*1*∆ under control and Cd stress conditions. Transcriptome analyses by RNA-Seq of WT and *pmk*-*1*∆ under control conditions (ctrl) and Cd stress (Cd) enabled **a** two stress-specific (WT_Cd_ vs. WT_ctrl_, *pmk*-*1*Δ_Cd_ vs. *pmk*-*1*Δ_ctrl_) (5-h incubations at 10 mmol/L CdCl_2_) and two strain-specific contrasts (WT_ctrl_ vs. *pmk*-*1*Δ_ctrl_, WT_Cd_ vs. *pmk*-*1*Δ_Cd_), which provided contrast-specific **b** numbers and **c** mean log_2_-fold changes (or differences) in expression (calculated using antilogarithmic data) of either all DEGs (*wide bars*) or of KOG-identified DEGs (*narrow bars*). Ratio values (*R*; *gray bars*) between **b** the numbers of up- and downregulated DEGs and **c** positive and negative mean log_2_-fold changes (or differences) were similar for DEGs and KOG-identified DEGs, and the changes in the *R* values between stress- or strain-specific contrasts (*dashed lines*) ran in opposite directions for **b** DEG numbers and **c** mean log_2_-fold changes (or differences)

To explore the role of PMK-1 p38 MAPK signaling in resistance against severe Cd stress at the genomic level, transcriptome profiling was carried out by RNA-Seq. We exposed WT or *pmk*-*1*Δ worms for 5 to 0 h (control, ctrl) or 10 mmol/L CdCl_2_ (Cd stress, Cd), with the 5-h incubation period guaranteeing an unaffected survival rate (see Fig. 1c). Transcriptome analyses resulted in two stress-specific and two strain-specific contrasts (Fig. 2a). The first stress-specific contrast (WT_Cd_ vs. WT_ctrl_) comprised 2659 up- and 1152 downregulated DEGs (defined by a false discovery rate, FDR <0.005), and the second stress-specific contrast (*pmk*-*1*Δ_Cd_ vs. *pmk*-*1*Δ_ctrl_) included 2607 up- and 785 downregulated DEGs (Fig. 2b; wide bars). To show exemplary expression changes of these DEGs, highly up- or downregulated DEGs from these contrasts [log_2_-fold changes >2 (i.e., 2^2^) or <−2 (i.e., 2^−2^)] with marked differences in expression between them (>2^1^) are listed in supplementary Table A1. Identification of the DEGs from the four contrasts via orthology (KOG identification; see the “Materials and methods” section) inevitably resulted in smaller numbers of KOG-identified DEGs (Fig. 2b; narrow bars). For all four contrasts, however, the ratios (*R*) between up- and downregulated genes were similar for DEGs and KOG-identified DEGs. This was true not only for DEG numbers but also for mean changes (stress-specific contrasts) and differences (strain-specific contrasts) in expression, specified as the mean log_2_-fold changes/differences (Fig. 2c). Thus, KOG-identified DEGs proved to be qualified representatives for all DEGs. Interestingly, there were contrary increases or decreases in *R* values for DEG numbers (Fig. 2b) or mean log_2_-fold changes/differences (Fig. 2c) between the two stress- or strain-specific contrasts (dashed lines), which may indicate alternative modes of regulation based on either the number of DEGs or expression changes/differences.

### Regulatory behavior of DEGs assigned to KOG categories

**Fig. 3:**
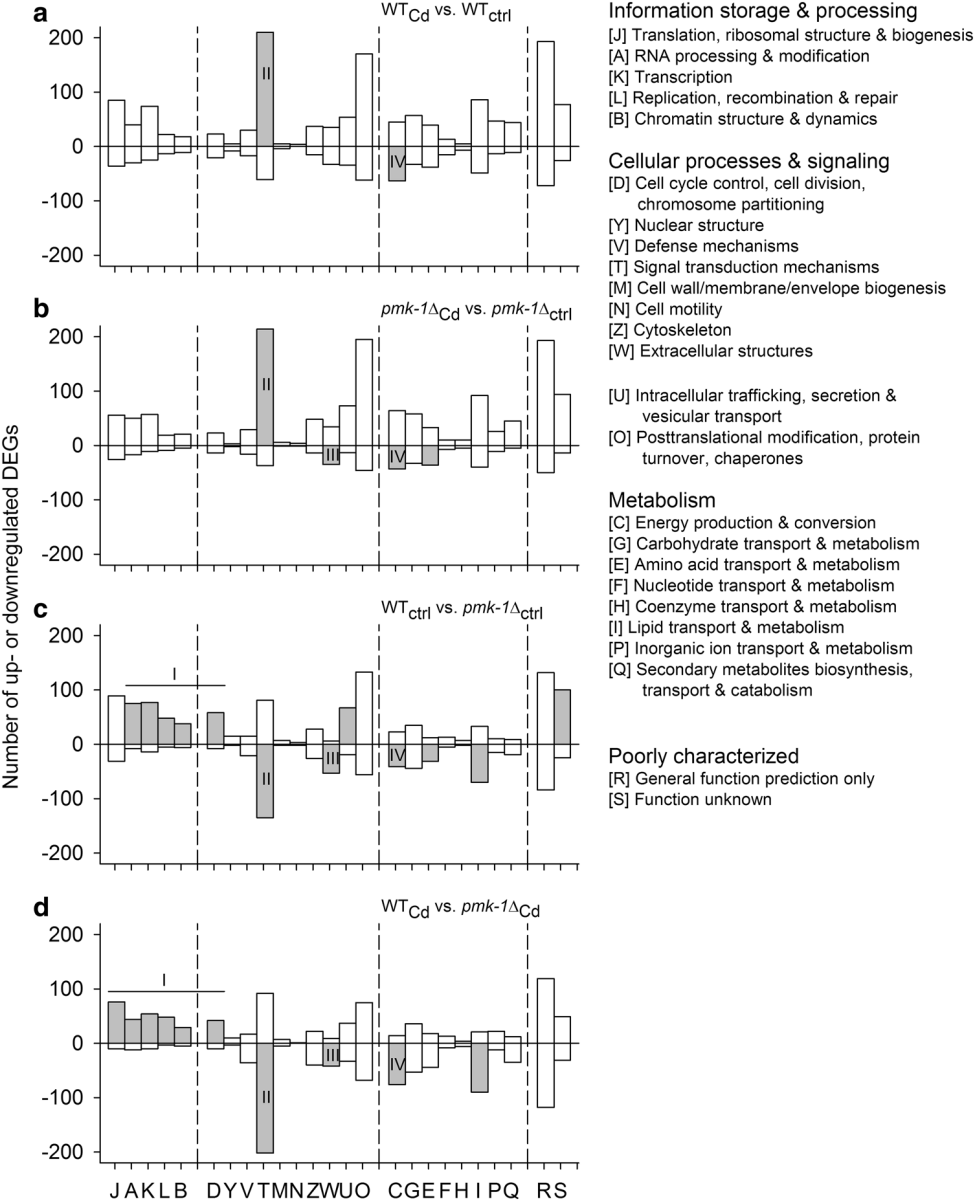
Functional assignments to KOG categories. KOG-identified DEGs from the (**a**, **b**) stress- and (**c**, **d**) strain-specific contrasts (see Fig. 2a) were classified into 25 superordinated KOG categories. Gene enrichment analyses (Chi-square tests; *P* < 0.001) identified KOG categories with deviating regulatory behavior (*gray bars*), for which the category-specific ratio between up- and downregulated DEGs was significantly different from the contrast-specific ratio. *Numerals I–IV* indicate the most prominent examples of deviating regulatory behavior

KOG-identified DEGs were classified for each contrast into 25 superordinated KOG categories (Fig. 3), and gene enrichment analyses (Chi-square analyses) were carried out to identify KOG categories with deviating regulatory behavior (gray bars).

In the case of strain-specific contrasts, five or six functionally related KOG categories (J, A, K, L, B, and D) with a focus on ‘DNA/chromosome, RNA, protein biosynthesis’ (Fig. 3c, d; I) contained an above-average number of upregulated DEGs (i.e., higher expression level in WT than in *pmk*-*1*∆). Furthermore, 354 (WT_ctrl_ vs. *pmk*-*1*Δ_ctrl_) or 265 (WT_Cd_ vs. *pmk*-*1*Δ_Cd_) of them could be annotated and functionally classified (functional annotation clustering) by DAVID 6.8. In the first case, 268 DEGs could be assigned to the GO term ‘developmental process’ (GO:0032502; ease score, e.s. = 3.5E−44), 175 to ‘gene expression’ (GO:0010467; e.s. = 1.6E−61) and 56 to ‘translation’ (GO:0006412; e.s. = 6.3E−31), with the latter group including many genes for ribosomal proteins, eukaryotic translation initiation factors, or tRNA ligases and synthetases. In the second case, 204 DEGs could be assigned to the GO term ‘developmental process’ (GO:0032502; e.s. = 4.4E−31), 133 to ‘gene expression’ (GO:0010467; e.s. = 5.0E−45) and 53 to ‘translation’ (GO:0006412; e.s. = 1.3E−33).

Significant deviations in regulatory behavior were also detected for the KOG category T (signal transduction mechanisms; II). In the stress-specific contrasts, the DEGs of this category comprised 35 (Fig. 3a) or 33 (Fig. 3b) upregulated genes for nuclear hormone receptors and 16 (Fig. 3a) or 10 (Fig. 3b) upregulated genes for C-type lectins and, in the strain-specific contrasts, 84 (Fig. 3c) or 121 (Fig. 3d) downregulated genes for phosphatases and protein kinases. Category W (extracellular structures; III) frequently contained an above-average number of downregulated DEGs, with 33 (Fig. 3b), 40 (Fig. 3c), or 27 (Fig. 3d) of them coding for collagens. There was also an above-average number of downregulated DEGs in category C (energy production and conversion; IV), with 23 (Fig. 3a), 17 (Fig. 3b), 11 (Fig. 3c), or 20 (Fig. 3d) of them coding for proteins involved in oxidative phosphorylation (David 6.8, KEGG pathway; e.s. <7.5E−12).

### Transcriptional regulation of SKN-1 target genes

**Fig. 4:**
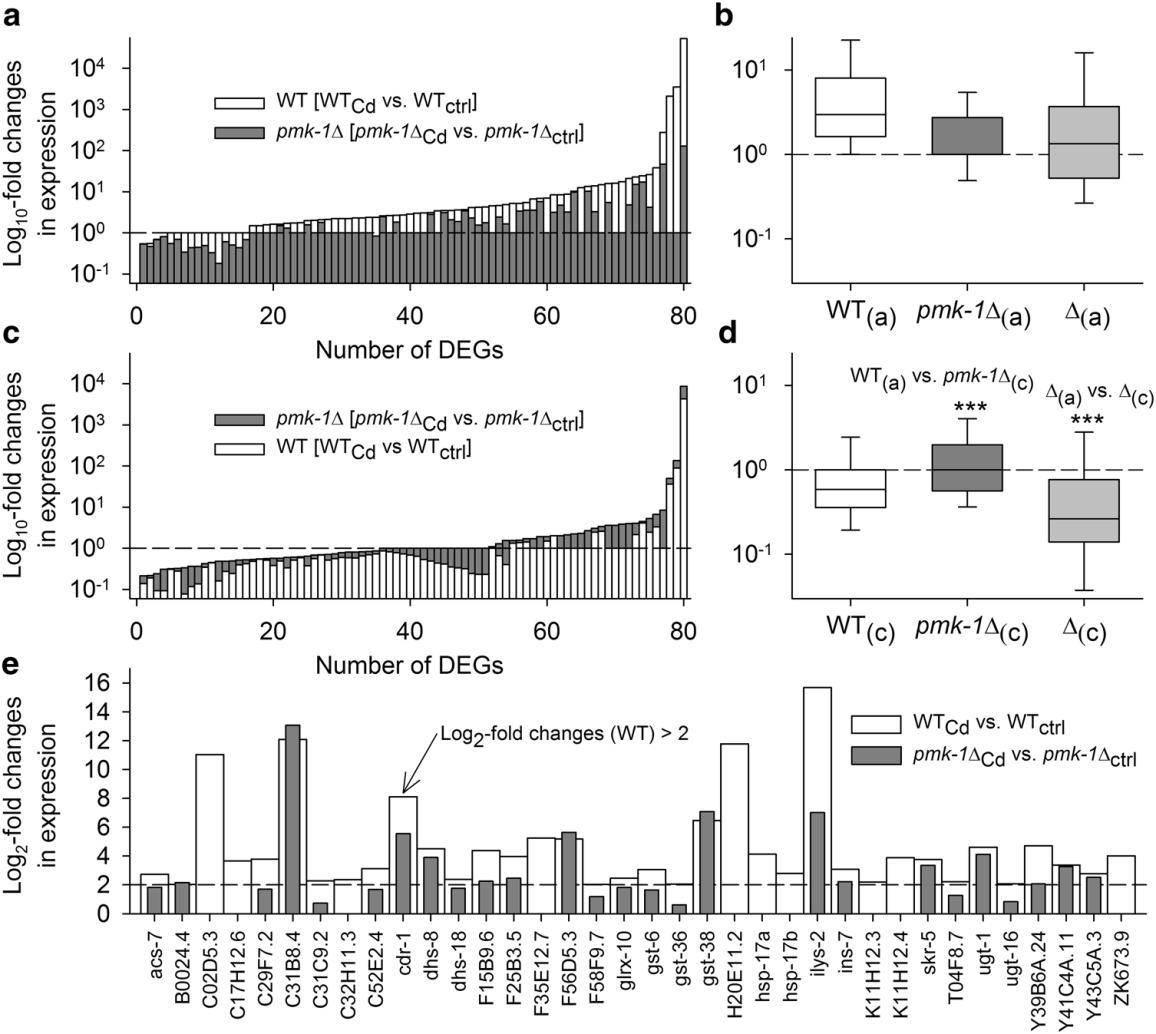
Expression of DEGs identified as SKN-1 target genes. DEGs from the stress-specific contrasts (see Fig. 2a) identified as SKN-1 target genes (Oliveira et al. 2009) were analyzed with regard to their absolute expression changes (only the graphs show them in a log presentation), thereby differentiating between the cases of **a** higher (i.e., more positive or less negative) expression changes in WT than in *pmk*-*1*∆ or **c** higher expression changes in *pmk*-*1*∆ than in WT (both 80 DEGs). Statistical analyses (Mann–Whitney rank-sum tests; ***P < 0.001) revealed significantly higher expression changes for WT in the first case (WT_(a)_; **b**) than for *pmk*-*1*∆ in the second one (*pmk*-*1*∆_(c)_; **d**) (the box plots show medians, 25th and 75th percentiles as box boundaries and 10th and 90th percentiles as *whiskers*). The differences in expression changes between WT and *pmk*-*1*∆ were also higher in the first case (∆_(a)_; **b**) than in the second one (∆_(c)_; **d**). **e** Mostly higher expression changes in WT than in *pmk*-*1*∆ were also found in the 36 DEGs (SKN-1 target genes) with strong differential expression in WT (log_2_-fold changes >2)

Aligning the DEGs from the stress-specific contrasts with SKN-1-regulated genes (control vs. *skn*-*1* RNAi-treated WT; Oliveira et al. 2009) resulted in 160 identical DEGs (supplementary Table A2), with 80 DEGs showing higher (i.e., more positive) Cd-induced expression changes in WT than in *pmk*-*1*∆ (Fig. 4a) and 80 more DEGs exhibiting the reverse regulatory behavior (Fig. 4c). Comparing the average changes in expression of WT in the first case (Fig. 4a) and *pmk*-*1*∆ in the second case (Fig. 4c) revealed significantly higher Cd-induced expression changes in WT than in *pmk*-*1*∆ (Fig. 4b, d; WT_(a)_ > *pmk*-*1*∆_(c)_). The difference in Cd-induced expression changes between both strains was also significantly higher in the first than in the second case (Fig. 4b, d; ∆_(a)_ > ∆_(c)_). Thus, although numbers of SKN-1-mediated genes with either higher or lower expression changes in WT than in *pmk*-*1*∆ were identical (80 DEGs each), Cd-induced expression changes proved to be generally higher in WT than in *pmk*-*1*∆. Several of the strongly upregulated DEGs (log_2_-fold changes >2 in WT; Fig. 4e; see also supplementary Table A2) are involved in (1) detoxification reactions and stress responses, including *cdr*-*1* and *glrx*-*10* as well as genes for glutathione *S*-transferases (C02D5.3, *gst* genes) and UDP-glucuronosyl/glucosyl transferases (*ugt* genes), (2) immunity and defense mechanisms (e.g., C17H12.6, F35E12.7, H20E11.2, *ilys*-*2*, ZK673.9), and (3) protein folding (*hsp*-*17*a,b).

### Transcriptional regulation of DAF-16 target genes

**Fig. 5:**
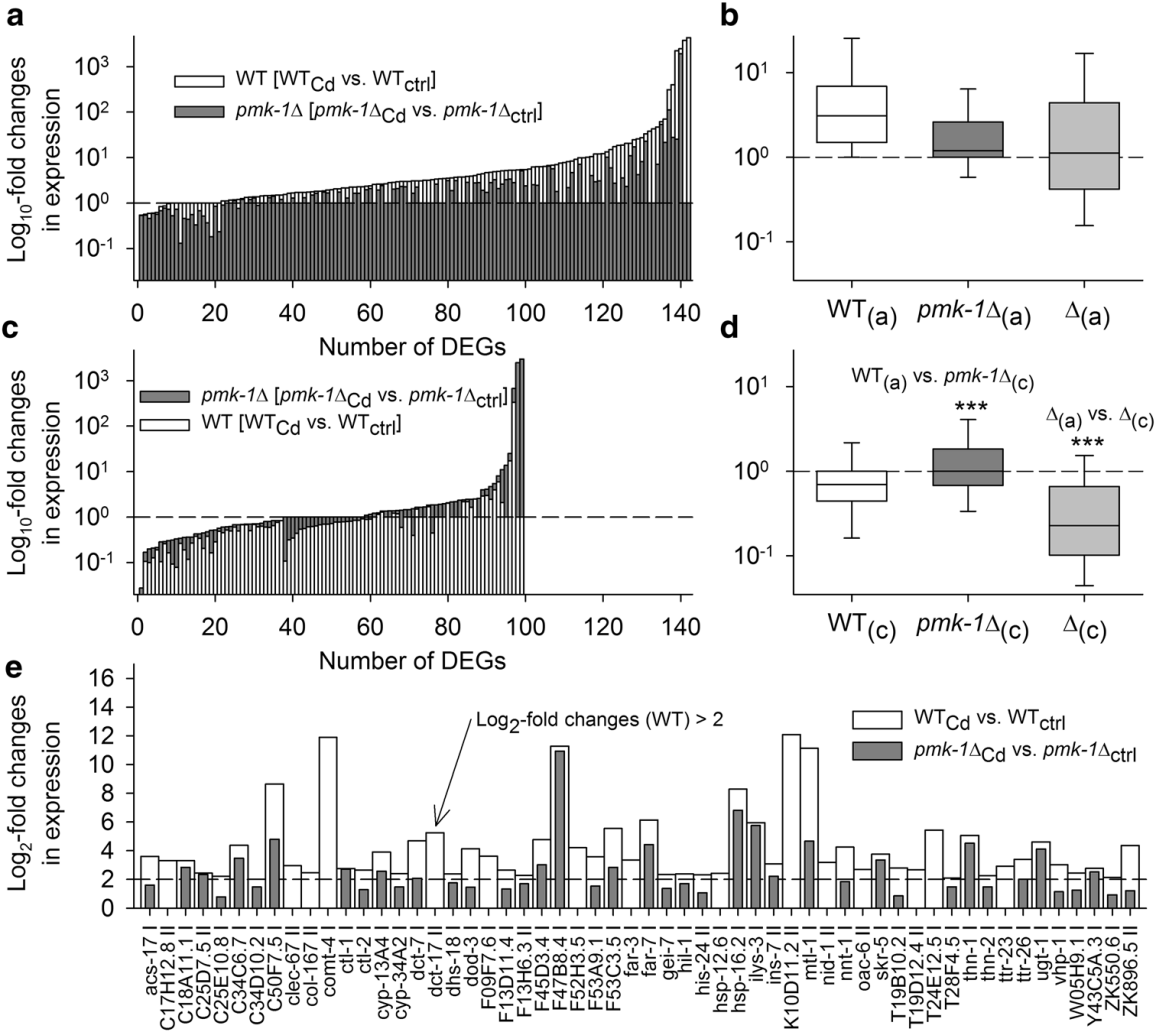
Expression of DEGs identified as DAF-16 target genes. DEGs from the stress-specific contrasts (see Fig. 2a) identified as DAF-16 target genes (Murphy et al. 2003) were analyzed in the same way as the DEGs identified as SKN-1 target genes (see Fig. 4). There were **a** 142 DEGs with higher (more positive or less negative) expression changes in WT than in *pmk*-*1*∆ and **c** 99 DEGs with higher expression changes in *pmk*-*1*∆ than in WT. Statistical analyses (Mann–Whitney rank-sum tests; ***P < 0.001) revealed significantly higher expression changes for WT in the first case (WT_(a)_; **b**) than for *pmk*-*1*∆ in the second one (*pmk*-*1*∆_(c)_; **d**). The differences in expression changes between WT and *pmk*-*1*∆ were also higher in the first case (∆_(a)_; **b**) than in the second one (∆_(c)_; **d**). **e** Mostly higher expression changes in WT than in *pmk*-*1*∆ were also found in the 56 DEGs (DAF-16 target genes) with strong differential expression in WT (log_2_-fold changes >2) (*symbols I* or *II* behind gene names denotes class I or II of DAF-16 target genes, according to the nomenclature of Murphy et al. 2003)

Aligning the DEGs from the stress-specific contrasts with DAF-16-regulated genes (upregulated class I or downregulated class II genes in *daf*-*2∆*; Murphy et al. 2003) resulted in 241 identical DEGs (supplementary Table A3). Here, 142 DEGs showed higher (more positive) Cd-induced expression changes in WT than in *pmk*-*1*∆ (Fig. 5a), and 99 more DEGs exhibited the reverse regulatory behavior (Fig. 5c). The average changes in expression in WT in the first case were significantly higher than those in *pmk*-*1*∆ in the second case (Fig. 5b, d; WT_(a)_ > *pmk*-*1*∆_(c)_). The difference between both strains in Cd-induced expression changes was also significantly higher in the first than in the second case (Fig. 5b, d; ∆_(a)_ > ∆_(c)_). Thus, there were more DAF-16-mediated genes with higher expression changes in WT than in *pmk*-*1*∆ than vice versa (142 vs. 99 DEGs), and, additionally, Cd-induced expression changes were generally higher in WT than in *pmk*-*1*∆. Several of the strongly upregulated DEGs (log_2_-fold changes >2 in WT; Fig. 5e; see also supplementary Table A3) code for catalase (*ctl*-*1*,*2*), cytochrome P450s (*cyp* genes), glutaredoxin 5 (F47B8.4), metallothionein (*mtl*-*1*), orthologs of human microsomal epoxide hydrolase 1, which converts toxic xenobiotics (K10D11.2, ZK896.5), and small heat shock proteins (*hsp*-*12.6*, *hsp*-*16.2*). The data thus showed that DAF-16-mediated gene expression levels were higher in the presence of PMK-1 (WT) and lower in the absence of PMK-1 (*pmk*-*1*∆).

### Interactions between PMK-1/SKN-1- and DAF-16-dependent gene control

**Fig. 6:**
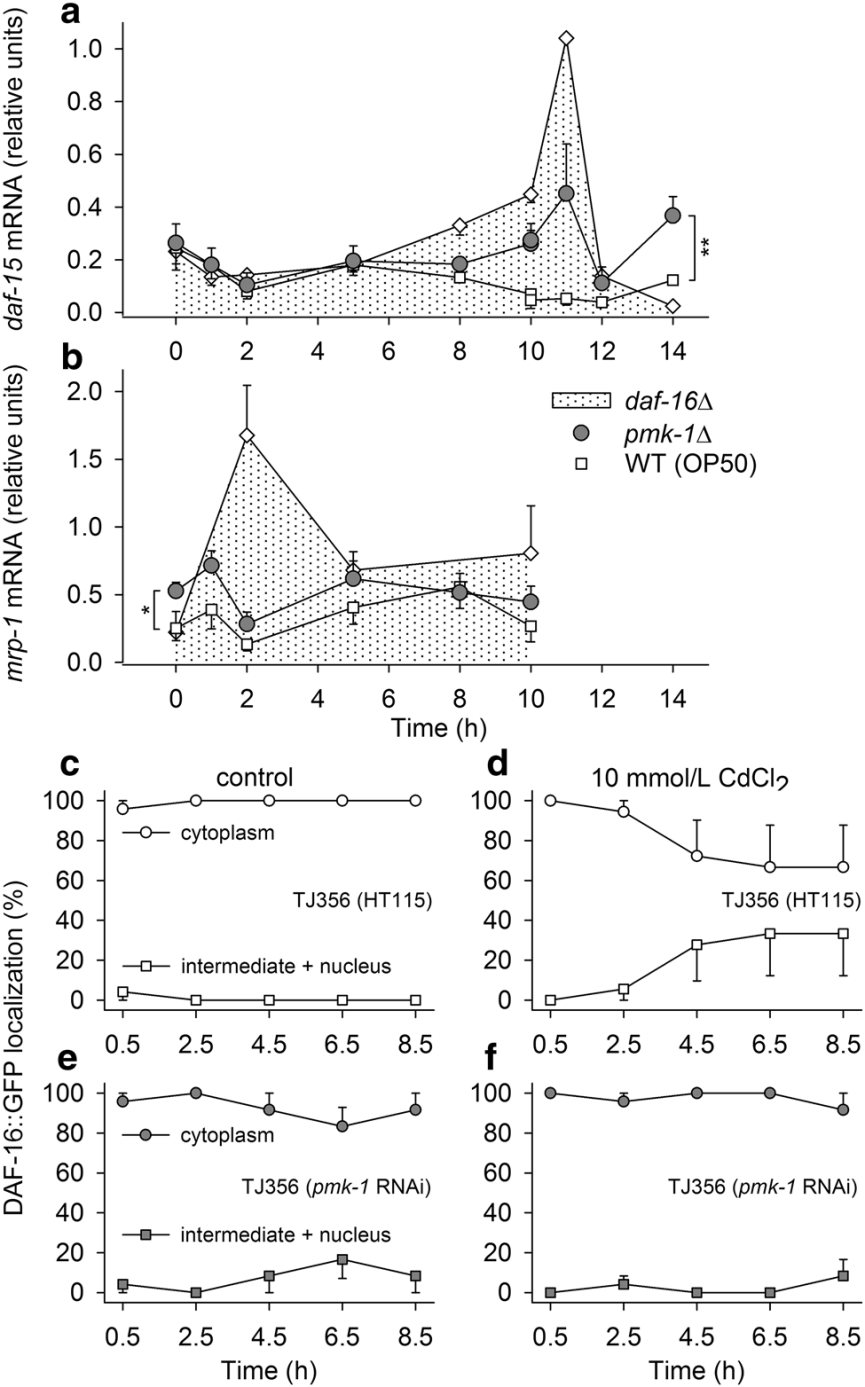
Temporal changes in *daf*-*15* and *mrp*-*1* mRNA levels (relative expression levels, with *cdc*-*42* mRNA as the reference) and DAF-16 subcellular localization under acute Cd stress (10 mmol/L CdCl_2_). Temporal changes in **a** *daf*-*15* or **b** *mrp*-*1* mRNA levels were measured in WT (OP50) (*squares*), *pmk*-*1*Δ (*circles*), and *daf*-*16*Δ (*diamonds*) under acute Cd stress (mean ± se, per mRNA type, point in time, and strain, *n* = 3–5 test groups with several hundred worms each). In addition, changes in the subcellular localization of DAF-16::GFP (cytoplasmic localization, *circles*; intermediate or nuclear localization, *squares*) were determined in the DAF-16::GFP expressing *C. elegans* strain TJ356 under **c**, **e** control conditions or **d**, **f** acute Cd stress in **c**, **d** control RNAi-(HT115) or **e**, **f** *pmk*-*1* RNAi-treated worms (mean ± se, per test condition, point in time, and RNAi treatment, *n* = 3 test groups with 4 worms each). *Asterisks* and *vertical bars* (in **a**, **b**) indicate overall differences between strains (two-way anova and Holm–Sidak’s method; *P < 0.05; **P < 0.01)

To gain a better understanding of the parallel decreases in PMK-1/SKN-1- and DAF-16-mediated gene expression in *pmk*-*1*∆ under Cd stress (Figs. 4, 5), we first measured the mRNA expression of the well-studied (Jia et al. 2004) negatively regulated DAF-16 target gene *daf*-*15*, which codes for the target of rapamycin complex 1 (TORC1) component raptor. *daf*-*15* mRNA expression was indeed highest in *daf*-*16*∆ after 11 h at 10 mmol/L CdCl_2_, followed by that in *pmk*-*1*Δ, and then by that in WT, which did not even upregulate *daf*-*15* expression (Fig. 6a). Thus, DAF-16-mediated effects (inhibited *daf*-*15* expression) were absent in *daf*-*16*∆, present in *pmk*-*1*Δ, but highest in WT. Interestingly, a similar result was found for the expression of the ABC transporter gene *mrp*-*1* (Fig. 6b), which indicates inhibition of *mrp*-*1* expression by nuclear DAF-16. DAF-16-binding motifs (two consensus DBE and five degenerate DAE motifs) are actually present in the sense and antisense strands of the (4 kb) upstream region of the *mrp*-*1* gene. Principles of the Cd-dependent induction of *daf*-*15* and *mrp*-*1* expression, however, are yet unknown.

Next, DAF-16 subcellular localization was determined in time-resolved experiments at 10 mmol/L CdCl_2_ using a control RNAi (HT115) or *pmk*-*1* RNAi-treated strain with DAF-16::GFP expression (TJ356). A slow Cd-induced DAF-16 response was detected in TJ356 (HT115) (Fig. 6d). In TJ356 (*pmk*-*1* RNAi), however, nuclear DAF-16 translocation was not detected in these experiments (Fig. 6f).

To further investigate PMK-1/SKN-1- and DAF-16-mediated gene expression, we quantified, under Cd stress (5 h at 10 mmol/L CdCl_2_), the mRNA level of genes that had previously been classified as SKN-1 (*nit*-*1*, *hsp*-*17*), DAF-16 (*ctl*-*2*, *hsp*-*12.6*), or SKN-1 and DAF-16 (*dhs*-*18*, *skr*-*5*, *ugt*-*1*) target genes (Murphy et al. 2003; Oliveira et al. 2009; Wang et al. 2010). These measurements were performed on control RNAi- (HT115) or *pmk*-*1* RNAi-treated WT and mutant strains, which expectedly differed in the stress-induced nuclear presence of SKN-1 (due to PMK-1 activation) and/or DAF-16: nuclear SKN-1 as well as nuclear DAF-16 [WT (HT115)]; essentially only nuclear DAF-16 [WT (*pmk*-*1* RNAi)]; only nuclear SKN-1 [*daf*-*16*∆ (HT115)]; no nuclear DAF-16 and essentially no nuclear SKN-1 [*daf*-*16*∆ (*pmk*-*1* RNAi)]; upregulated nuclear DAF-16 level and essentially no nuclear SKN-1 [*daf*-*2*∆ (*pmk*-*1* RNAi)].

**Fig. 7:**
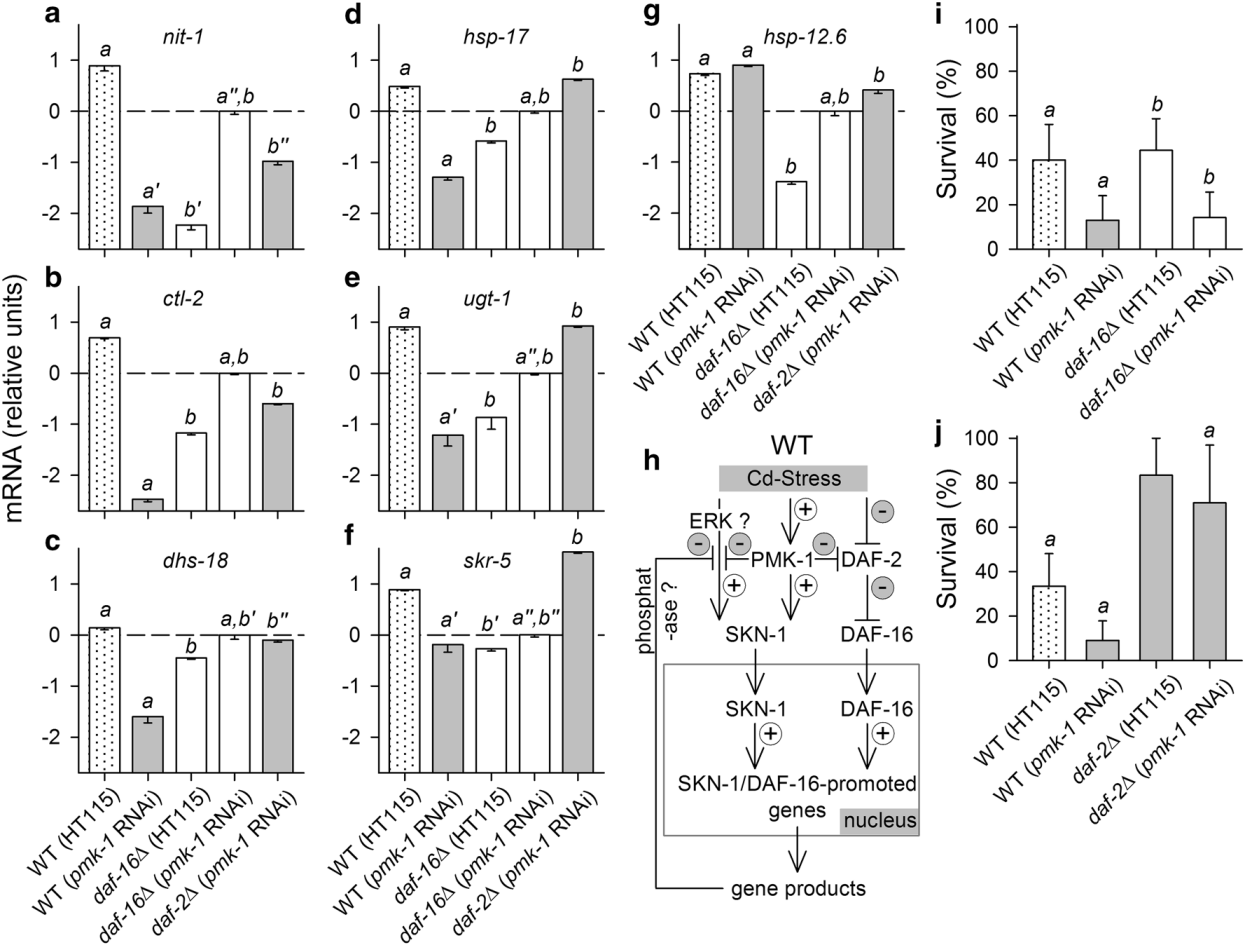
Expression of SKN-1 and DAF-16 target genes and survival under Cd stress. The mRNA level (relative expression levels, with *cdc*-*42* mRNA as the reference) of genes, previously classified as SKN-1, DAF-16, or SKN-1 and DAF-16 target genes (Murphy et al. 2003; Oliveira et al. 2009; Wang et al. 2010), was measured under Cd stress (5 h at 10 mmol/L CdCl_2_) using RT-qPCR: **a** *nit*-*1*, **b** *ctl*-*2*, **c** *dhs*-*18*, **d** *hsp*-*17*, **e** *ugt*-*1*, **f** *skr*-*5*, and **g** *hsp*-*12.6*. Measurements were carried out on control RNAi- (HT115) or *pmk*-*1* RNAi-treated WT and mutants: WT (HT115), WT (*pmk*-*1* RNAi), *daf*-*16*∆ (HT115), *daf*-*16*∆ (*pmk*-*1* RNAi), and *daf*-*2*∆ (*pmk*-*1* RNAi). Two independent experimental series were performed on WT (HT115), WT (*pmk*-*1* RNAi), and *daf*-*16*∆ (*pmk*-*1* RNAi) on the one hand and *daf*-*16*∆ (HT115), *daf*-*2*∆ (*pmk*-*1* RNAi), and again *daf*-*16*∆ (*pmk*-*1* RNAi) on the other, with the mRNA levels of *daf*-*16*∆ (*pmk*-*1* RNAi) used for normalization (*dashed horizontal lines*, mRNA level = 10^0^). Note that logarithmic (log_10_) scales were used to also show very low mRNA levels (mean ± se, per mRNA type and test animal group, *n* = 6–8 measurements on several hundred worms each). **h** Based on these and other data, a model of the relationships between PMK-1 and SKN-1- and DAF-16-dependent gene control under Cd stress was derived (see “Discussion” for details). Additionally, the survival rates of the differently treated strains were determined upon long-lasting Cd stress (24 h at 10 mmol/L CdCl_2_) in **i**, **j** two independent experimental series (mean ± SD, per test animal group, *n* = 12–24 measurements on 9–22 worms each). (*Dotted bars*, WT (HT115); *white bars*, *daf*-*16*∆; *gray bars*, others.) Statistically significant differences (*P* < 0.05; one-way anova and Holm–Sidak’s method) within each of the two experimental series are indicated by the *letters a* or *b* (*simple letters*, significant differences between all test animal groups of one experimental series; *letters with line marks*, significant differences only to the group marked with a *simple letter*)

WT (HT115) frequently expressed the highest mRNA quantities and WT (*pmk*-*1* RNAi) or *daf*-*16*∆ (HT115) the lowest ones (Fig. 7a–f), which indicates that both transcription factors contribute to the promotion of gene expression. *daf*-*2*∆ (*pmk*-*1* RNAi) showed increasing mRNA levels in the order of the genes *nit*-*1*, *ctl*-*2*, *dhs*-*18*, *hsp*-*17*, *ugt*-*1*, and *skr*-*5* (Fig. 7a–f). WT (*pmk*-*1* RNAi) followed a similar trend, which suggests an increasing impact of DAF-16 on the expression of these genes. *hsp*-*12.6* mRNA levels were high in animal groups, which are characterized by the presence of DAF-16 [WT (HT115), WT (*pmk*-*1* RNAi), and *daf*-*2*∆ (*pmk*-*1* RNAi)] (Fig. 7g). In contrast to previous expectations, *daf*-*16*∆ (*pmk*-*1* RNAi) took an intermediate position in gene expression, which may be caused by another stronger activation factor of SKN-1 other than PMK-1 (see “Discussion”). As these results indicate that, in most cases (Fig. 7a–f), SKN-1 as well as DAF-16 promoted gene expression, with the mRNA level depending on the nuclear quantity and specific impact of SKN-1 and DAF-16, we searched for SKN-1 and DAF-16 binding motifs nearby these genes (2 kb upstream or downstream, intragenic regions) and actually found such motifs in all cases (Table 2).

**Table 2:** SKN-1 and DAF-16 binding motifs detected 2 kb upstream from start codons, 2 kb downstream from stop codons, and in intragenic regions with EMBOSS fuzznuc (Rice et al. 2000). SKN-1 motif: RTCAT (R = G/A) (Rupert et al. 1998); DAF-16 motifs: DBE (DAF-16 binding element), TRTTTAC (R = G/A); DAE (DAF-16 associated element), CTTATCA (Murphy 2006)

Gene nameName	Gene symbol	Number of SKN-1 motifs			Total	Number of DAF-16 motifs (DBE, DAE)			Total
		−2 kb	intragenic	+2 kb		−2 kb	intragenic	+2 kb	
ZK1058.6	*nit*-*1*	5	0	3	8	1	0	0	1
Y54G11A.5	*ctl*-*2*	3	0	1	4	3	1	2	6
C45B11.3	*dhs*-*18*	4	0	3	7	1	0	0	1
F52E1.7	*hsp*-*17*	6	0	2	8	2	0	1	3
AC3.7	*ugt*-*1*	3	0	2	5	3	1	1	5
F47H4.10	*skr*-*5*	2	0	6	8	2	0	1	3
F38E11.2	*hsp*-*12.6*	3	0	5	8	2	0	6	8
	Total	26	0	22	48	14	2	11	27

In a concluding study on these strains, we tested for the final outcome of long-lasting Cd stress (Fig. 7i, j). After 24 h at 10 mmol/L CdCl_2_, the survival rate of WT (*pmk*-*1* RNAi) or *daf*-*16*∆ (*pmk*-*1* RNAi) was significantly lower and that of *daf*-*2*∆ (*pmk*-*1* RNAi) significantly higher than that of control RNAi-treated WT or *daf*-*16*∆.

### Transcriptional regulation of genes for molecular chaperones, ABC transporters, and other stress-responsive/protective proteins

**Fig. 8:**
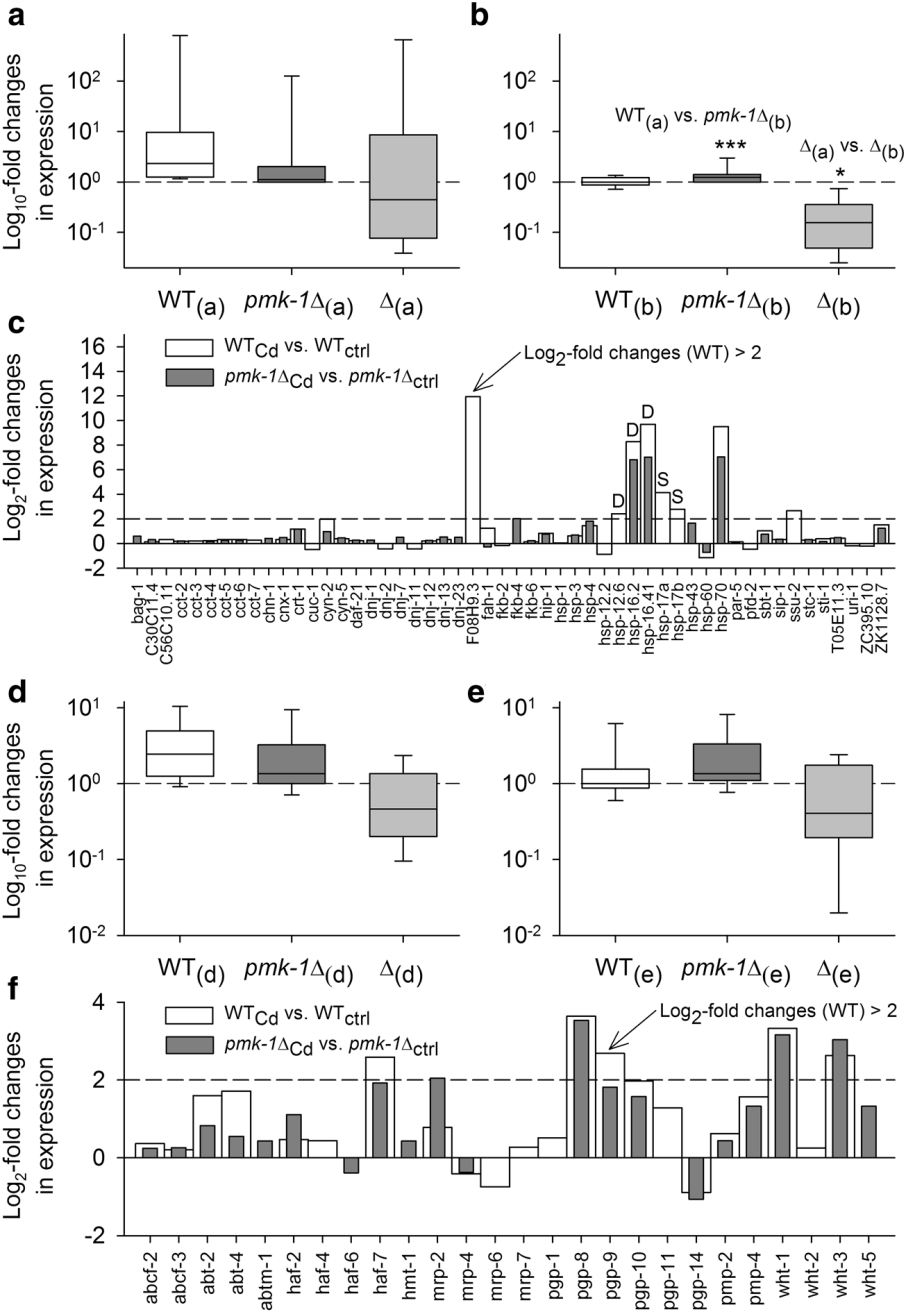
Expression of DEGs for chaperones and ABC transporters. DEGs for chaperones and ABC transporters from the stress-specific contrasts (see Fig. 2a) were analyzed in the same way as the DEGs identified as SKN-1 target genes (see Fig. 4). With regard to genes for chaperones, there were 22 DEGs with higher (more positive or less negative) expression changes in WT than in *pmk*-*1*∆ and 30 DEGs with higher expression changes in *pmk*-*1*∆ than in WT. Statistical analyses (Mann–Whitney rank-sum tests; ***P < 0.001; *P < 0.05) revealed significantly higher expression changes for WT in the first case (WT_(a)_; **a**) than for *pmk*-*1*∆ in the second one (*pmk*-*1*∆_(b)_; **b**), and the differences in the expression changes between WT and *pmk*-*1*∆ were also higher in the first case (∆_(a)_; **a**) than in the second one (∆_(b)_; **b**). **c** Mostly higher expression changes in WT than in *pmk*-*1*∆ were also found in the eight DEGs (for chaperones) with strong differential expression in WT (log_2_-fold changes >2) (*symbols D* or *S* indicate DEGs, previously classified as DAF-16 or SKN-1 target genes). With respect to genes for ABC transporters, there were 17 DEGs with higher expression changes in WT than in *pmk*-*1*∆ and 9 DEGs with higher expression changes in *pmk*-*1*∆ than in WT. **d**, **e** There were no significant differences in expression changes. **f** Five DEGs (for ABC transporters) showed high differential expression in WT (log_2_-fold changes >2)

Analyzing the stress-specific contrasts specifically for genes encoding molecular chaperones revealed 52 DEGs (Fig. 8c), with 22 of them showing higher (more positive) Cd-induced expression changes in WT than in *pmk*-*1*∆ and 30 DEGs exhibiting reverse regulatory behavior. The average changes in the expression levels in WT in the first case were higher than those in *pmk*-*1*∆ in the second case (Fig. 8a, b; WT_(a)_ > *pmk*-*1*∆_(b)_). From the eight DEGs showing high expression changes in WT (log_2_-fold changes >2; Fig. 8c), three genes coding for small heat shock proteins (α-crystallins) are known DAF-16 target genes (D) (Murphy et al. 2003; Halaschek-Wiener et al. 2005). Three more genes also code for small heat shock proteins (*hsp*-*17*a,b; F08H9.3), with two of them likely being SKN-1 target genes (S). The gene *hsp*-*70* codes for an inducible heat shock protein, and *ssu*-*2* is a predicted molecular chaperone containing a DnaJ domain. Related to HSPs, the metal-inducible gene *numr*-*1* (not graphically represented), whose gene product co-localizes with heat shock factor 1 (HSF-1) as a possible component of nuclear stress granules (Tvermoes et al. 2010), was highly but similarly upregulated in WT and *pmk*-*1*Δ under Cd stress (log_2_-fold changes, 7.53 vs. 7.85).

Screening the stress-specific contrasts for genes encoding ABC transporters revealed 26 DEGs (Fig. 8f), with 17 of them showing higher (more positive) Cd-induced expression changes in WT than in *pmk*-*1*∆ and 9 DEGs exhibiting reverse regulatory behavior. There were no significant differences in the average changes in expression (Fig. 8d, e). Most DEGs coded for the P-glycoprotein family of ABC transporters (Fig. 8f).

Focusing on Cd-induced expression changes of known Cd-responsive/protective genes, measured by RNA-Seq first and then by RT-sqPCR, showed much higher expression changes of the Cd-responsive gene *ttm*-*1*, which codes for a cation diffusion facilitator protein (Huffman et al. 2004; Roh et al. 2013), and metallothionein genes (*mtl*-*1*, *mtl*-*2*) in WT than in *pmk*-*1*Δ (Table 3). Moreover, Cd-induced expression changes differed clearly between PMK-1, SKN-1, or DAF-16 target genes (*cdr*-*1*, *ttm*-*1*, *mtl*-*1*, *mtl*-*2*) and genes activated by other transcription factors (*hmt*-*1*, *mrp*-*1*, *pcs*-*1*, *pgp*-*1*), with the latter genes coding for proteins involved in the active export of toxins out of cells (ABC transporters). Upregulated DEGs in the stress-specific contrasts also included genes for the three-phase detoxification system of eukaryotes (e.g., Sarkadi et al. 2006), which involved 27 *cyp* genes, 31 *gst* genes, and 27 *ugt* genes (data not shown). From the already known KGB-1 MAPK target genes under heavy metal stress (Hattori et al. 2013), both *kreg*-*1* and *kreg*-*2* were upregulated in the contrast WT_Cd_ vs. WT_ctrl_ and only *kreg*-*2* was upregulated in the contrast *pmk*-*1*∆_Cd_ vs. *pmk*-*1*∆_ctrl_.

**Table 3:** Log_2_-fold changes in mRNA expression (u, unchanged expression) of known metal/Cd-responsive/protective genes (gene name, gene symbol) in the case of stress-specific contrasts, determined by RNA-Seq and partly also by RT-sqPCR (mean ± se, per mRNA type, test condition, and strain, *n* = 3–5 test groups with several hundred worms each)

Gene name	Gene symbol	Control	WT_Cd_ vs. WT_ctrl_ (RNA-Seq)	*pmk*-*1*Δ_Cd_ vs. *pmk*-*1*Δ_ctrl_ (RNA-Seq)	WT_Cd_ vs. WT_ctrl_ (RT-sqPCR)	*pmk*-*1*Δ_Cd_ vs. *pmk*-*1*Δ_ctrl_ (RT-sqPCR)
K11G9.6	*mtl*-*1*	DAF-16 (+)	11.13	4.67	10.10 ± 0.97 (*n* = 4)	4.01 ± 0.48 (*n* = 4)
F35E8.11	*cdr*-*1*	SKN-1 (+)	8.11	5.54		
Y39E4A.2b	*ttm*-*1*	PMK-1 (+)	4.04	1.80	3.48 ± 0.67 (*n* = 3)	1.09 ± 0.04 (*n* = 3)
T08G5.10	*mtl*-*2*	DAF-16 (+)	3.84	1.05	1.43 ± 0.11 (*n* = 3)	1.06 ± 0.02 (*n* = 3)
K08E7.9	*pgp*-*1*		0.52	u		
W09D6.6	*hmt*-*1*		u	0.43		
F57C12.5	*mrp*-*1*	DAF-16 (−)	u	u	1.61 (data from Fig. 6b)	1.17 (data from Fig. 6b)
F54D5.1	*pcs*-*1*		u	u		

Known or identified positive (+) or negative (−) control factors are PMK-1, SKN-1, and DAF-16

## Discussion

The present study focused on the roles of important components of stress-signaling pathways and of ABC transporters for the resistance of *Caenorhabditis elegans* to Cd stress.

### Survival under Cd stress

To activate the full stress response spectrum, the effects of Cd stress were studied at the upper tolerance limit of *C. elegans* (Fig. 1), with 10 mmol/L CdCl_2_ proving to be a test condition at which the survival rates fell below the 50 percent mark after 24 h. This kind of Cd stress has already previously been applied (Barsyte et al. 2001; Roh et al. 2009). The present study revealed PMK-1 p38 mitogen-activated protein kinase (MAPK) signaling to be involved in Cd resistance mechanisms, as a *pmk*-*1* mutant (*pmk*-*1*∆) and *pmk*-*1* RNAi-treated WT showed reduced and a *pmk*-*1*-overexpressing strain (PMK-1::GFP) showed elevated survival rate under Cd stress in comparison to that observed in WT. The result that each change of insulin-like (DAF-2) signaling promoted Cd resistance of the respective strains (*daf*-*2*Δ, *daf*-*16*Δ, or DAF-16::GFP) will be discussed below. With regard to ABC transporters, which translocate a wide variety of substrates across membranes, *mrp*-*1* mutation evidently reduced Cd resistance.

### Transcriptomics

These results raise new questions concerning interactions and targets of cellular signal processing systems. The present study focused on these questions by first studying and analyzing large-scale gene expression profiles by RNA-Seq (Figs. 2, 3). To avoid significant adverse effects on health and survival but to still induce severe stress, the worms were exposed for only 5 h (see Fig. 1c) to 10 mmol/L CdCl_2_ in the RNA-Seq experiments. Differentially expressed genes (DEGs) were studied in strain- and stress-specific contrasts, involving four comparisons between stressed or non-stressed WT and *pmk*-*1*∆. The functional description of these DEGs, which was carried out by means of orthology (KOG database), was a prerequisite for a more detailed analysis. Therefore, we ensured that regulatory behavior was similar between the entirety of the DEGs and the smaller group of KOG-identified DEGs. Functional classification into the 25 superordinated KOG categories and gene enrichment analysis were used to identify general differences in gene expression patterns between stressed or non-stressed WT and *pmk*-*1*Δ.

### PMK-1 activity in relation to protein biosynthesis

The strain-specific contrasts (Fig. 3c, d) revealed positive effects of PMK-1 (in WT) on gene expression for protein biosynthesis. Promoting effects of PMK-1 on these genes were also reported in a study on the function of PMK-1 in *C. elegans* under heat stress (Mertenskötter et al. 2012). In mammals and *Drosophila*, Cully et al. (2010) showed promoting effects of p38 MAPK on TOR (TORC1) signaling which positively regulates protein biosynthesis and growth and promotes gene expression for these processes (Wullschleger et al. 2006; Urban et al. 2007; Huber et al. 2011). However, additional experiments of the present study (Fig. 6a) suggested the existence of a compensatory mechanism in the case of reduced TORC1 activation (*pmk*-*1*∆). A gene (*daf*-*15*) coding for the essential TORC1 component raptor was significantly higher upregulated in *pmk*-*1*∆ than in WT after several hours of Cd stress. Thus, elevated gene expression for TORC1 complexes may compensate in the medium term for the reduced TORC1 activation in *pmk*-*1*∆. A compensation for reduced TORC1 activity by an expression of TORC1 genes has already previously been reported (Robida-Stubbs et al. 2012). The expression of *daf*-*15* is negatively controlled by DAF-16 (Jia et al. 2004). Thus, the decrease in the *daf*-*15* mRNA level from *daf*-*16*∆, over *pmk*-*1*∆, to WT can be explained by the absence of DAF-16 in *daf*-*16*∆ and a lower nuclear level of DAF-16 in *pmk*-*1*∆, which matches the results obtained in *pmk*-*1* RNAi-treated TJ356 worms (Fig. 6f).

### PMK-1 activity in relation to DAF-16-dependent gene control

PMK-1 and DAF-16 have previously been suggested to independently but concurrently control genes involved in immune responses (Troemel et al. 2006). Under Cd stress, however, PMK-1 promoted DAF-16-mediated gene expression and DAF-16 nuclear localization (Figs. 5, 6d). There are several ways how PMK-1 may affect DAF-16. One of them would be a control of extracellular signals (insulin-like peptides). There is evidence for a neuronal MAPK/JNK-1-dependent modulation of peripheral DAF-2 signaling via insulin-like peptides (Wolf et al. 2008). As PMK-1 is also present in neurons (Mertenskötter et al. 2012), neuronal PMK-1 might influence the secretion of insulin-like peptides. A decrease in activating ILPs or an increase in inhibitory ILPs would raise the nuclear DAF-16 level (by lowering DAF-2 signaling) and initiate DAF-16-mediated gene expression. Alternatively, PMK-1 may directly promote DAF-16 nuclear translocation (e.g., by inhibiting DAF-2 signaling in the cell). Independent from mechanism, the positive effects of PMK-1 on DAF-16 nuclear localization and DAF-16-mediated gene expression are clearly advantageous, as they enhance the Cd resistance of WT (see below).

Differing from regulations under Cd stress, however, heat stress caused a higher upregulation of DAF-16-mediated stress genes in *pmk*-*1*∆ than in WT (e.g., genes for small heat shock proteins; Mertenskötter et al. 2012). Thus, regulatory directions can change under different types of stress, which requires another mode of promoting DAF-16 nuclear translocation. Heat stress has immediate negative effects on cells and likely endangers cellular proteins more than Cd stress, which may result in a higher activity of SEK-1 (i.e., a MAP2K of the PMK-1 pathway) under heat stress than under Cd stress. In case of present PMK-1, nuclear translocation of PMK-1::GFP was observed under heat stress (Mertenskötter et al. 2012) but not under Cd stress (R. J. Paul, unpublished data) in a transgenic strain. Furthermore, heat shock factor 1 (HSF-1)-controlled chaperone genes (e.g., *hsp*-*1*, *hsp*-*60*, *cct* and *dnj* genes) were specifically upregulated under heat stress (Mertenskötter et al. 2012) but not under Cd stress (Fig. 8c) in WT. In case of absent PMK-1 (*pmk*-*1*∆), however, elevated SEK-1 activity may be redirected towards the SEK-1-dependent promotion of DAF-16 nuclear translocation and DAF-16-mediated gene expression that has already been reported (Kondo et al. 2005; Mertenskötter et al. 2012).

### PMK-1 activity in relation to SKN-1 and DAF-16-dependent gene control

Experiments were performed to determine the mRNA level of several genes in Cd-stressed control or *pmk*-*1* RNAi-treated WT and mutant strains (Fig. 7a–g). Cd-induced gene expression was elevated if both transcription factors were effective [WT (HT115)] and reduced if only one transcription factor was effective [WT (*pmk*-*1* RNAi), *daf*-*16*∆ (HT115)]. The mRNA level probably increased in WT (*pmk*-*1* RNAi) and *daf*-*2*∆ (*pmk*-*1* RNAi) with a rising impact of DAF-16 on gene expression. The elevated *hsp*-*12.6* expression in WT (HT115), WT (*pmk*-*1* RNAi), and *daf*-*2*∆ (*pmk*-*1* RNAi) indicates a particularly high impact of DAF-16 on the expression of this gene (Murphy et al. 2003) with a neglectable effect of nuclear DAF-16 quantity. These results suggest that the expression of these genes depended on both nuclear quantity and specific impact of SKN-1 and DAF-16. Analyzing the putative control regions of these genes revealed SKN-1 as well as DAF-16 binding motifs in all cases (Table 2).

The intermediate mRNA level in *daf*-*16*∆ (*pmk*-*1* RNAi) is, however, difficult to explain. Possibly, an emergency reaction takes place under severe stress if essential stress-signaling components are absent (DAF-16) or not activated by PMK-1 (SKN-1). Alternatively, the ERK-MAPK pathway might cause an increased SKN-1 activation (Okuyama et al. 2010; Blackwell et al. 2015). This would require, however, an inhibition of the ERK pathway by the PMK-1 pathway (e.g., via negative cross-talk) as well as by DAF-16 target gene products (e.g., cell signaling phosphatases) to exclude positive ERK effects on SKN-1 activation in *daf*-*16*∆ (HT115) or WT (*pmk*-*1* RNAi), where Cd-induced gene expression was reduced. We have integrated the hypothetical functioning of the ERK pathway in a model, which shows the relationships between PMK-1 and SKN-1- and DAF-16-dependent gene control under Cd stress (Fig. 7h). However, functions and regulation of SKN-1 are remarkably complex (Blackwell et al. 2015). Therefore, we have not included the known inhibition of SKN-1 by signaling components of the DAF-2 pathway (i.e., inactivation of SKN-1 by AKT-1, -2, and SGK-1; Tullet et al. 2008), because DAF-2 signaling was always deactivated in our experiments either by mutation [*daf*-*2*∆ (*pmk*-*1* RNAi)] or by the Cd stress applied. For similar reasons, this model also does not take into account the MAP2K SEK-1, which might play a role during the heat stress response (see above).

Somewhat deviating from the measured mRNA levels after 5 h of severe Cd stress (10 mmol/L CdCl_2_), the survival rate after 24 h of severe Cd stress (Fig. 7i, j) was certainly highest in *daf*-*2*∆, which indicates a particular importance of high nuclear DAF-16 levels for a long-term survival under Cd stress. The reduced survival rates of *pmk*-*1* RNAi-treated WT and *daf*-*16*∆ shows that PMK-1 signaling is also important for a long-term survival under Cd stress. However, it should also be noted that the seven genes, whose expression was determined after 5 h of Cd stress, are not necessarily essential for a long-term survival under this stress condition.

### PMK-1 activity in relation to other cellular processes

A number of Cd-induced DEGs of the KOG category T (signal transduction mechanisms) (Fig. 4a, b) code for nuclear hormone receptors, which are activated by lipophilic (steroid) hormones (and xenobiotics as well) to control metabolism, development and homeostasis (Antebi 2006). Cd might have positively affected their expression by mimicking effects of lipophilic hormones via high affinity binding to corresponding receptors (Johnson et al. 2003). The expression of immune response genes (genes for C-type lectins in KOG category T) will be discussed below. Particularly in strain-specific contrasts (Fig. 4c, d), there were several KOG categories (e.g., categories T or W, extracellular structures) with a significant surplus of downregulated DEGs (i.e., upregulated DEGs in *pmk*-*1*Δ in comparison to WT). This concerned in particular DEGs for phosphatases and protein kinases in category T, which may represent some form of compensation or substitution for the absent PMK-1 signaling in *pmk*-*1*Δ. The upregulated expression of collagen genes in category W in *pmk*-*1*Δ in comparison to WT may result from oxidative stress, which has been suggested to stimulate collagen synthesis (Liao and Freedman 1998).

### Cd stress resistance and SKN-1 target genes

PMK-1 (in WT) enhanced Cd stress resistance, inter alia, by activating the transcription factor SKN-1 and promoting the expression of SKN-1 target genes (Fig. 4). SKN-1-mediated genes with higher Cd-induced expression changes in WT than in *pmk*-*1*∆ included *cdr*-*1* (Liao et al. 2002) or *gst* and *ugt* genes for glutathione S-transferases and UDP-glucuronosyl/glucosyl transferases, which conjugate glutathione (GSTs) or glucuronic acid (UGTs) to a variety of different substrates, including Cd (see Marrs 1996 for the GSTs), peroxidized lipids, or xenobiotics, for their transport out of cells (Sarkadi et al. 2006; Burmeister et al. 2008). Genes for members of the *hsp*-*16*/*hsp*-*20*/α-crystallin family of small heat shock proteins (*hsp*-*17*a,b) were also upregulated more intensely in WT than in *pmk*-*1*∆.

### Cd stress resistance and DAF-16 target genes

Reduced or inhibited DAF-2 signaling confers stress resistance via elevated DAF-16 nuclear occupancy. Consequently, *daf*-*2*Δ as well as the DAF-16-overexpressing strain TJ356 showed higher Cd stress resistance. PMK-1 (in WT) additionally promoted the expression of DAF-16 target genes (Fig. 5). Among them were *ctl*-*2* coding for the H_2_O_2_ scavenger catalase (Storey 1996) or genes for small heat shock proteins (*hsp*-*12.6*, *hsp*-*16.2*) and metallothionein (*mtl*-*1*). Other DAF-16 target genes (identified by previous studies; e.g., Halascheck-Wiener et al. 2005) with higher Cd-induced expression changes in WT than in *pmk*-*1*Δ included *hsp*-*16.41* (encodes a member of the *hsp*-*16*/*hsp*-*20*/αB-crystallin family) and *mtl*-*2*. Genes for metallothioneins are already known to be upregulated under Cd stress (Freedman et al. 1993; Cioci et al. 2000; Barsyte et al. 2001). Metallothioneins are able to bind Cd in vitro and in vivo (Zeitoun-Ghandour et al. 2010), and mutation or RNAi of one or both *C. elegans mtl* genes was reported to confer hypersensitivity to Cd stress (at concentrations above 75 µmol/L) when testing for brood size and lifespan (Swain et al. 2004; Hughes and Stürzenbaum 2007). A recent study, however, suggested that *mtl*-*1* and *mtl*-*2* (and even *cdr*-*1*) are not required for resistance against metal toxicity during developmental processes, which was explained by a compensatory increase in phytochelatins (Hall et al. 2012).

### Cd stress resistance and genes for chaperones

To protect and repair the protein equipment of the cell, stress-specific strategies of chaperone control seem to be pursued, which are possibly related to the risk and/or type of protein damage under different types of stress. Heat stress caused in WT a PMK-1-dependent upregulation of genes for the HSF-1-controlled chaperone machinery (Mertenskötter et al. 2012), whereas Cd stress induced in WT an upregulation of genes for primarily small heat shock proteins (Fig. 8c). Small heat shock proteins (HSP-12.6, HSP-16.2, HSP-16.41, F08H9.3, HSP-17a,b), of which DAF-16 or SKN-1 control has been reported elsewhere (e.g., Murphy et al. 2003; Oliveira et al. 2009), prevent unfolded proteins from aggregating (Leroux et al. 1997). Positive effects of Cd on the expression of *hsp*-*16* or *hsp*-*70*-encoded heat shock proteins (as shown in this study) have also been reported (David et al. 2003; Cui et al. 2007). Thus, an elevated Cd-induced expression of genes for primarily small heat shock proteins further increased the Cd resistance of WT.

### Cd stress resistance and genes for ABC transporters

The observed reduced Cd resistance of the ABC transporter mutant *mrp*-*1*∆ is in accordance with a previous study (Broeks et al. 1996) showing developmental processes negatively affected by moderate Cd stress (80 µmol/L CdCl_2_) in a *mrp*-*1* mutant. Gene expression for ABC transporters was not influenced by PMK-1 (Fig. 8d, e). Consequently, other control mechanisms must exist for their expression. The expression of *mrp*-*1* was found to be negatively controlled by DAF-16 (Fig. 6b). This mechanism might explain the elevated Cd resistance of *daf*-*16*Δ, as a Cd-induced increase in *mrp*-*1* expression could have improved the Cd resistance of *daf*-*16*Δ (cf., Winter et al. 2016). The result of reduced *mrp*-*2* mRNA and MRP-2::DsRed fusion protein levels in *daf*-*2*Δ or *daf*-*2* RNAi-treated worms in comparison to control worms (R. J. Paul, unpublished data) shows that *mrp*-*2* expression is also negatively controlled by DAF-16. The negative relationship between DAF-16 and *mrp*-*1*/*mrp*-*2* expression likely originates from processes related to dauer diapause. DAF-16 supports the formation of dauer larvae (Hu 2007), which are covered by a special cuticle that occludes all orifices of the worm. Excretory gland cell function seems to be arrested during the dauer stage as secretory granules are lacking (Riddle and Albert 1997). Thus, the secretion of waste products via MRP-1/MRP-2 activity likely ceases in dauer larvae, which could explain the DAF-16-mediated downregulation of *mrp*-*1*/*mrp*-*2* expression. Yabe et al. (2005) have already suggested a link between insulin-like signaling and MRP-1 as well as an inhibitory function of MRP-1 for dauer larva formation (i.e., MRP-1-mediated export of a dauer-inducing substance).

### Cd stress resistance and known Cd-responsive/protective genes

The distinct difference in Cd-induced expression changes between two groups of known Cd-responsive/protective genes, with the PMK-1, SKN-1, or DAF-16 target genes *ttm*-*1*, *cdr*-*1*, and *mtl*-*1/2* highly inducible by Cd stress and the genes for ABC transporters and helper proteins (*hmt*-*1*, *mrp*-*1*, *pgp*-*1*, *pcs*-*1*) rather constitutively expressed (Table 3), indicates different functions during the Cd stress response. The first group possibly codes for a rapid Cd trap-and-efflux system, whereas proteins of the second group may be better suited for long-term moderate Cd stress, possibly due to a more restricted transport rate of these ABC transporters. Compensatory high PCS-1 activity (phytochelatin synthesis) in *cdr*-*1* and *mtl*-*1/2* mutants as well as the relative insensitivity of these mutants to moderate Cd stress in the micromolar range (Hall et al. 2012) supports the suggestion of different operating ranges of Cd-responsive/protective proteins.

### Cd stress resistance and immunity

Providing immunity against pathogenic attack seems to be the ancestral function of p38 MAPK signaling (Troemel et al. 2006; Bolz et al. 2010). However, PMK-1 is also involved in other types of stress response (see “Introduction”), including the response to Cd stress as shown in the present study. The multitude of PMK-1-mediated stress responses entails a Cd-induced expression of immune response genes (supplementary Tables A2, A3; see also Cui et al. 2007), including genes for C-type lectins or cytochrome P450s. C-type lectins are secreted carbohydrate-binding proteins functioning as recognition tool for antimicrobial defense (e.g., Schulenburg et al. 2008). Cytochrome P450s function as oxidoreductases (monooxygenases) to improve the solubility of hydrophobic endobiotics/xenobiotics for their export out of cells (phase 1 reaction during biotransformation; Menzel et al. 2001). The broad-spectrum effect of PMK-1 signaling against abiotic and biotic stressors likely has biological relevance, as it may prevent deadly infections, for instance, when the worm is suffering from Cd stress in its terrestrial habitat.

## Conclusion

*pmk*-*1* mutation or RNAi caused a severely reduced Cd resistance, which was due to the downregulation of several stress response mechanisms. The absent PMK-1-mediated activation of the transcription factor SKN-1 resulted in a reduced expression of SKN-1 target genes, which normally serve for antioxidant defense and detoxification systems. The parallel reduction in DAF-16 target gene expression further reduced the stress resistance of the *pmk*-*1* mutant, which lowered, for instance, gene expression for molecular chaperones under Cd stress. PMK-1 (in WT), in turn, promoted gene expression for components of the protein biosynthesis apparatus, matching the elevated stress response activity in WT. MRP-1 improved the Cd resistance of wild-type but in contrast to DAF-16, PMK-1 did not affect the expression of ABC transporter genes. PMK-1- and DAF-16-dependent gene control also influenced the expression of immune genes under Cd stress. Thus, PMK-1 regulates SKN-1 and DAF-16 activity at least under Cd stress, affects TORC1 signaling and protein biosynthesis, and seems to provide double protection against abiotic and biotic stressors.

## Electronic supplementary material

Below is the link to the electronic supplementary material.

Supplementary material 1 (DOCX 61 kb)

## Abbreviations

ABC transporters: ATP-binding cassette transporters protein superfamily
AKT-1/-2: AKT kinase family 1/2
CCT: Chaperonin-containing TCP-1 family
CDR-1: cadmium-responsive protein 1
CYP: Cytochrome P450
DAF-2: Abnormal dauer formation family protein 2
DAF-15: Abnormal dauer formation family protein 15
DAF-16: Abnormal dauer formation family protein 16
DEG: Differentially expressed gene
DNJ: DNaJ domain family
FoxO: Forkhead box O subclass proteins
GST: Glutathione *S*-transferase
HMT-1: Heavy metal tolerance factor 1
HSF-1: Heat shock factor 1
JNK-1: c-Jun N-terminal Kinase 1
KGB-1: Kinase, GLH-binding protein 1
kreg: KGB-1 regulated gene
MAPK: Mitogen activated protein kinase
MAP2K: MAPK kinase
MRP-1: Multidrug resistance family protein 1
MTL-1: Metallothionein protein 1
MTL-2: Metallothionein protein 2
PCS-1: Phytochelatin synthase 1
PGP-1: P-Glycoprotein 1
PMK-1: P38 map kinase family protein 1
RAPTOR: Regulatory-associated protein of mTOR
RNA-Seq: RNA sequencing
ROS: Reactive oxygen species
SEK-1: SAPK/ERK kinase 1
SGK-1: Serum- and glucocorticoid-inducible kinase homolog
SKN-1: SKiNhead protein 1
TTM-1: Toxin-regulated targets of MAPK
mTORC1: Mechanistic target of rapamycin complex 1
WT: Wild type
