# Supplementary material for: PMK-1 p38 MAPK promotes cadmium stress resistance, the expression of SKN-1/Nrf and DAF-16 target genes, and protein biosynthesis in Caenorhabditis elegans
Source: Mol Genet Genomics. 2017 Aug 1;292(6):1341–61. doi: 10.1007/s00438-017-1351-z (PMC5682872; doi:10.1007/s00438-017-1351-z)
Supplement: Supplementary file 1 — Supplementary material 1 (DOCX 61 kb) [file 438_2017_1351_MOESM1_ESM.docx]

**(Electronic supplementary) Table A1.** DEGs showing high expression changes (log_2_-fold changes > 2 or < -2) in the contrasts WT_Cd_ vs. WT_ctrl_ (WT in the table) and *pmk-1*∆_Cd_ vs. *pmk-1*∆_ctrl_ (*pmk-1*∆ in the table), with a log_2_-fold difference between both contrasts of at least 1 (= 2^1^).

| **Gene** | **Function** | **WT** | ***pmk-1*∆** | **Gene** | **Function** | **WT** | ***pmk-1*∆** |
| --- | --- | --- | --- | --- | --- | --- | --- |
|  |  | Log_2_-fold changes | |  |  | Log_2_-fold changes | |
| F48C1.9 | hypothetical protein | 17.34 | 3.59 | ZK1307.2 | hypothetical protein | 4.57 | 14.69 |
| *ilys-2* | Invertebrate LYSozyme | 15.68 | 7.02 | *nlp-34* | Neuropeptide-Like Protein | 5.29 | 14.22 |
| C54F6.5 | hypothetical protein | 13.87 | 2.96 | *cyp-13A8* | Putative cytochrome P450 CYP13A8 | 6.31 | 14.19 |
| F19B10.13 | hypothetical protein | 13.81 | 4.55 | *nlp-30* | QWGYGGY-amide | 4.11 | 13.30 |
| C25F9.11 | hypothetical protein | 13.62 | 4.49 | C26F1.1 | hypothetical protein | 2.92 | 13.27 |
| F19G12.10 | hypothetical protein | 13.60 | 2.55 | *clec-121* | C-type LECtin | 10.97 | 11.98 |
| *lips-11* | LIPaSe related | 12.51 | 7.42 | Y34D9A.8 | hypothetical protein | 4.13 | 11.40 |
| F22E5.6 | hypothetical protein | 12.51 | 4.61 | K10H10.10 | hypothetical protein | 4.31 | 11.34 |
| *sdz-35* | SKN-1 Dependent Zygotic transcript | 12.47 | 6.18 | Y9C9A.1 | hypothetical protein | 3.76 | 10.84 |
| EGAP1.1 | hypothetical protein | 12.28 | 3.09 | *acd-1* | hypothetical protein | 3.37 | 10.11 |
| *try-5* | TRYpsin-like protease | 12.26 | 4.01 | *fbxa-163* | F-box A protein | 8.39 | 9.40 |
| *sri-36* | Serpentine Receptor, class I | 12.02 | 10.81 | T28A11.19 | hypothetical protein | 3.32 | 6.41 |
| C30H6.12 | hypothetical protein | 11.93 | 4.32 | *pho-6* | intestinal acid PHOsphatase | 3.43 | 5.77 |
| *bath-46* | BTB and MATH domain containing | 11.45 | 4.89 | Y102A11A.9 | hypothetical protein | 3.71 | 5.29 |
| *tsp-4* | Tetraspanin | 11.41 | 4.87 | W03G1.5 | hypothetical protein | 4.07 | 5.29 |
| *fbxa-66* | F-box A protein | 11.21 | 4.72 | T20D4.10 | hypothetical protein | 2.73 | 5.21 |
| *mtl-1* | Metallothionein-1 | 11.13 | 4.67 | *irg-2* | Infection Response Gene | 3.95 | 5.20 |
| ZK1240.1 | hypothetical protein | 10.96 | 4.25 | *tbb-6* | TuBulin, Beta | 2.32 | 4.74 |
| *fbxa-48* | F-box A protein | 10.95 | 3.36 | *oac-53* | O-ACyltransferase homolog | 2.21 | 4.66 |
| *pqn-29* | Prion-like-(Q/N-rich)-domain-bearing protein | 10.93 | 3.96 | Y82E9BL.18 | hypothetical protein | 3.62 | 4.66 |
| *math-8* | MATH (meprin-associated Traf homology) domain containing | 10.70 | 4.82 | *cpg-7* | Chondroitin proteoglycan 7 | 3.46 | 4.63 |
| *cyp-13A6* | Putative cytochrome P450 CYP13A6 | 10.01 | 4.21 | K08B5.1 | hypothetical protein | 2.71 | 4.55 |
| *hsp-16.41* | Heat shock protein Hsp-16.41 | 9.69 | 7.03 | C52A10.1 | Carboxylic ester hydrolase | 3.14 | 4.33 |
| *hsp-70* | Heat Shock Protein | 9.50 | 7.04 | C10C5.2 | hypothetical protein | 2.92 | 4.25 |
| *nmr-1* | NMDA class glutamate Receptor | 9.14 | 3.87 | *cyp-14A4* | CYtochrome P450 family | 2.84 | 4.21 |
| C50F7.5 | hypothetical protein | 8.64 | 4.79 | C05D9.9 | hypothetical protein | 2.81 | 4.20 |
| M03A1.8 | hypothetical protein | 8.30 | 3.48 | *lys-3* | Lysozyme-like protein 3 | 2.57 | 4.13 |
| *hsp-16.2* | Heat Shock Protein | 8.28 | 6.81 | F14F9.4 | hypothetical protein | 2.64 | 4.09 |
| *cdr-1* | CaDmium Responsive | 8.11 | 5.54 | T02B11.4 | hypothetical protein | 2.52 | 3.87 |
| R05H10.1 | hypothetical protein | 7.53 | 2.92 | *fbxa-37* | F-box A protein | 2.42 | 3.76 |
| C25F9.2 | hypothetical protein | 7.41 | 4.74 | *flp-21* | FMRF-Like Peptide | 2.48 | 3.55 |
| T10C6.15 | hypothetical protein | 7.40 | 5.38 | F56D2.5 | hypothetical protein | 2.15 | 3.30 |
| F08G2.5 | hypothetical protein | 6.79 | 5.00 | R03H10.7 | hypothetical protein | 2.25 | 3.26 |
| C18H7.1 | hypothetical protein | 6.65 | 3.21 | *cyp-32A1* | CYtochrome P450 family | 2.11 | 3.24 |
| Y53F4B.25 | hypothetical protein | 6.55 | 3.87 | *piit-1* | Protease Inhibitor I2 (TWO) | -3.86 | -2.04 |
| *oac-14* | O-ACyltransferase homolog | 6.47 | 2.41 | F58G6.3 | hypothetical protein | -3.42 | -2.06 |
| *cpt-3* | Carnitine Palmitoyl Transferase | 6.45 | 4.06 | *lbp-8* | Lipid Binding Protein | -4.24 | -2.06 |
| T26H5.9 | hypothetical protein | 6.31 | 4.83 | *acp-6* | ACid Phosphatase family | -3.73 | -2.09 |
| *far-7* | Fatty Acid/Retinol binding protein | 6.14 | 4.42 | *clec-1* | C-type LECtin | -3.69 | -2.17 |
| F53B2.8 | hypothetical protein | 6.14 | 4.29 | C35A5.3 | hypothetical protein | -4.22 | -2.20 |
| F10D7.3 | Uncharacterized monothiol glutaredoxin F10D7.3 | 6.02 | 2.51 | *cyp-29A4* | CYtochrome P450 family | -4.33 | -2.26 |
| M60.7 | hypothetical protein | 5.91 | 3.98 | *col-93* | COLlagen | -3.55 | -2.29 |
| F56D6.8 | hypothetical protein | 5.90 | 2.84 | T12B5.14 | hypothetical protein | -3.86 | -2.40 |
| *dct-8* | DAF-16/FOXO Controlled, germline Tumor affecting | 5.88 | 2.83 | *elo-6* | Elongation of very long chain fatty acids protein 6 | -3.99 | -2.43 |
| ZK105.5 | hypothetical protein | 5.88 | 4.36 | *col-179* | COLlagen | -3.63 | -2.48 |
| F33H12.7 | hypothetical protein | 5.68 | 3.51 | *dhs-25* | DeHydrogenases, Short chain | -4.17 | -2.60 |
| *nlp-31* | QWGYGGY-amide | 5.63 | 4.42 | *gcsh-1* | Glycine Cleavage System H protein | -4.41 | -2.62 |
| F53C3.5 | hypothetical protein | 5.55 | 2.84 | *vit-1* | Vitellogenin-1 | -4.30 | -2.72 |
| T22B7.3 | hypothetical protein | 5.44 | 3.80 | T13F3.6 | hypothetical protein | -5.58 | -2.88 |
| C49G7.7 | hypothetical protein | 5.41 | 3.49 | *ugt-63* | UDP-GlucuronosylTransferase | -4.62 | -3.38 |
| *cpt-4* | Carnitine Palmitoyl Transferase | 5.34 | 3.65 | T28D6.3 | hypothetical protein | -5.98 | -3.76 |
| *ptr-22* | PaTched Related family | 5.31 | 4.21 | *col-43* | COLlagen | -4.95 | -3.79 |
| D2063.1 | hypothetical protein | 5.30 | 2.25 | F08D12.2 | hypothetical protein | -13.11 | -4.99 |
| C06B3.7 | hypothetical protein | 5.29 | 4.05 | *nas-3* | Zinc metalloproteinase nas-3 | -12.51 | -5.19 |
| ZK6.8 | hypothetical protein | 5.26 | 2.59 |  |  |  |  |
| K09D9.1 | hypothetical protein | 5.25 | 3.04 |  |  |  |  |
| C39B5.14 | hypothetical protein | 5.19 | 3.80 |  |  |  |  |
| *zip-10* | bZIP transcription factor family | 5.17 | 3.69 |  |  |  |  |
| *cyp-33C7* | CYtochrome P450 family | 5.02 | 3.56 |  |  |  |  |
| K05B2.4 | hypothetical protein | 4.96 | 2.49 |  |  |  |  |
| T05E7.4 | hypothetical protein | 4.88 | 3.39 |  |  |  |  |
| *tre-5* | Trehalase | 4.84 | 2.52 |  |  |  |  |
| H09F14.1 | hypothetical protein | 4.84 | 2.90 |  |  |  |  |
| F45D3.4 | hypothetical protein | 4.77 | 3.02 |  |  |  |  |
| *asp-17* | ASpartyl Protease | 4.70 | 2.07 |  |  |  |  |
| *dct-7* | DAF-16/FOXO Controlled, germline Tumor affecting | 4.69 | 2.08 |  |  |  |  |
| F56C11.6 | Carboxylic ester hydrolase | 4.65 | 3.09 |  |  |  |  |
| *cyp-13A7* | Putative cytochrome P450 CYP13A7 | 4.62 | 3.16 |  |  |  |  |
| Y62F5A.9 | hypothetical protein | 4.62 | 3.61 |  |  |  |  |
| *ftn-1* | Ferritin | 4.55 | 2.03 |  |  |  |  |
| Y15E3A.5 | hypothetical protein | 4.55 | 2.45 |  |  |  |  |
| Y53C12B.7 | hypothetical protein | 4.51 | 2.33 |  |  |  |  |
|  |  | 4.41 | 2.21 |  |  |  |  |
| C13A10.2 | hypothetical protein | 4.40 | 2.99 |  |  |  |  |
| F15B9.6 | hypothetical protein | 4.39 | 2.26 |  |  |  |  |
| *col-121* | COLlagen | 4.37 | 2.65 |  |  |  |  |
| *slc-17.9* | SLC (SoLute Carrier) homolog | 4.33 | 2.16 |  |  |  |  |
| *fbl-1* | FiBuLin | 4.31 | 2.42 |  |  |  |  |
| *pha-4* | Defective pharyngeal development protein 4 | 4.31 | 2.82 |  |  |  |  |
| W02A2.9 | hypothetical protein | 4.23 | 2.74 |  |  |  |  |
| *faah-2* | Fatty Acid Amide Hydrolase homolog | 4.20 | 2.48 |  |  |  |  |
| F37A8.5 | Protein yippee-like F37A8.5 | 4.17 | 2.57 |  |  |  |  |
| *swt-1* | Sugar transporter SWEET1 | 4.16 | 2.04 |  |  |  |  |
| *oac-31* | O-ACyltransferase homolog | 4.16 | 2.09 |  |  |  |  |
| Y41G9A.10 | hypothetical protein | 4.09 | 2.64 |  |  |  |  |
| *dao-6* | Dauer or Aging adult Overexpression | 4.06 | 3.05 |  |  |  |  |
| M01G12.14 | hypothetical protein | 4.06 | 2.16 |  |  |  |  |
| *ikb-1* | I Kappa B homolog | 4.05 | 2.14 |  |  |  |  |
| *ghi-1* | Glycoprotein Hormone (FLR-2) Interacting protein | 4.05 | 2.50 |  |  |  |  |
| *nmur-4* | NMUR (NeuroMedin U Receptor) homolog | 4.05 | 2.35 |  |  |  |  |
| Y41D4B.6 | hypothetical protein | 3.99 | 2.93 |  |  |  |  |
| F25B3.5 | hypothetical protein | 3.98 | 2.46 |  |  |  |  |
| F43D9.1 | hypothetical protein | 3.96 | 2.15 |  |  |  |  |
| *lact-5* | beta-LACTamase domain containing | 3.95 | 2.13 |  |  |  |  |
| *nhr-180* | Nuclear Hormone Receptor family | 3.93 | 2.81 |  |  |  |  |
| ZC443.3 | hypothetical protein | 3.92 | 2.59 |  |  |  |  |
| *cebp-1* | C/EBP (CCAAT/enhancer-binding protein) homolog | 3.92 | 2.86 |  |  |  |  |
| *cyp-13A4* | Putative cytochrome P450 CYP13A4 | 3.90 | 2.57 |  |  |  |  |
| *dpy-14* | DumPY: shorter than wild-type | 3.90 | 2.12 |  |  |  |  |
| *scl-2* | SCP-Like extracellular protein | 3.81 | 2.62 |  |  |  |  |
| *siah-1* | E3 ubiquitin-protein ligase siah-1 | 3.80 | 2.20 |  |  |  |  |
| *hch-1* | Zinc metalloproteinase nas-34 | 3.80 | 2.37 |  |  |  |  |
| F59B2.12 | hypothetical protein | 3.80 | 2.02 |  |  |  |  |
| F47B8.2 | hypothetical protein | 3.69 | 2.35 |  |  |  |  |
| F46A8.7 | hypothetical protein | 3.63 | 2.05 |  |  |  |  |
| Y57G11B.2 | hypothetical protein | 3.59 | 2.08 |  |  |  |  |
| T19C4.1 | hypothetical protein | 3.57 | 2.55 |  |  |  |  |
| M01A8.1 | hypothetical protein | 3.46 | 2.22 |  |  |  |  |
| Y37H2A.14 | hypothetical protein | 3.42 | 2.31 |  |  |  |  |
| *dsl-3* | Delta-like protein | 3.40 | 2.35 |  |  |  |  |
| *ttr-26* | TransThyretin-Related family domain | 3.40 | 2.02 |  |  |  |  |
| *nhr-206* | Nuclear Hormone Receptor family | 3.29 | 2.04 |  |  |  |  |
| *nhr-44* | Nuclear hormone receptor family member nhr-44 | 3.24 | 2.05 |  |  |  |  |
| *nhr-133* | Nuclear hormone receptor family member nhr-133 | 3.07 | 2.01 |  |  |  |  |
| F18E3.12 | hypothetical protein | -2.42 | -6.45 |  |  |  |  |
| F46F2.3 | hypothetical protein | -4.23 | -5.83 |  |  |  |  |
| Y48E1B.8 | hypothetical protein | -6.04 | -7.29 |  |  |  |  |

**(Electronic supplementary) Table A2.** SKN-1 target genes, with columns showing (from left to right) gene number, gene name, gene symbol, symbol explanation, additional information, and log_2_-fold changes in expression (WT_Cd_ vs. WT_ctrl_ and *pmk-1*∆_Cd_ vs. *pmk-1*∆_ctrl_). Gray-shaded cells indicate more positive or less negative Cd-induced expression changes in *pmk-1*∆ than in WT.

| **Number** | **Gene name** | **Gene symbol** | **Symbol explanation** | **Additional information** | **WT_Cd_ vs. WT_ctrl_** | ***pmk-1*∆_Cd_ vs. *pmk-1*∆_ctrl_** |
| --- | --- | --- | --- | --- | --- | --- |
| 1 | C45G7.2 | *ilys-2* | Invertebrate lysozyme |  | 15.68 | 7.02 |
| 2 | C31B8.4 |  |  |  | 12.08 | 13.06 |
| 3 | H20E11.2 |  |  | Involved in innate immune response | 11.78 | 0.00 |
| 4 | C02D5.3 |  |  | *gsto-2*, glutathione S-transferase, omega class | 11.04 | 0.00 |
| 5 | F35E8.11 | *cdr-1* | Cadmium responsive |  | 8.11 | 5.54 |
| 6 | F35E8.8 | *gst-38* | Glutathione S-transferase |  | 6.47 | 7.07 |
| 7 | F35E12.7 |  |  | *dct-7*, involved in innate immune response | 5.25 | 0.00 |
| 8 | F56D5.3 |  |  | FMN binding activity and oxidoreductase activity | 5.18 | 5.65 |
| 9 | Y39B6A.24 |  |  | *asp-17*, aspartyl protease | 4.70 | 2.07 |
| 10 | AC3.7 | *ugt-1* | UDP-glucuronosyltransferase |  | 4.61 | 4.12 |
| 11 | K10H10.3 | *dhs-8* | Dehydrogenases, short chain |  | 4.51 | 3.91 |
| 12 | F15B9.6 |  |  |  | 4.39 | 2.26 |
| 13 | F52E1.7 | *hsp-17a* | Heat shock protein |  | 4.13 | 0.00 |
| 14 | ZK673.9 |  |  | *clec-143*, C-type lectin | 4.01 | 0.00 |
| 15 | F25B3.5 |  |  |  | 3.98 | 2.46 |
| 16 | K11H12.4 |  |  |  | 3.88 | 0.00 |
| 17 | C29F7.2 |  |  |  | 3.80 | 1.69 |
| 18 | F47H4.10 | *skr-5* | Skp1 related (ubiquitin ligase complex component) |  | 3.76 | 3.36 |
| 19 | C17H12.6 |  |  | Involved in innate immune response | 3.65 | 0.00 |
| 20 | Y41C4A.11 |  |  | Beta' (beta-prime) subunit of the coatomer (COPI) complex | 3.37 | 3.27 |
| 21 | C52E2.4 |  |  |  | 3.13 | 1.69 |
| 22 | ZK1251.2 | *ins-7* | Insulin related |  | 3.07 | 2.23 |
| 23 | F11G11.3 | *gst-6* | Glutathione S-transferase |  | 3.06 | 1.65 |
| 24 | F52E1.7 | *hsp-17b* | Heat shock protein |  | 2.80 | 0.00 |
| 25 | Y43C5A.3 |  |  |  | 2.77 | 2.52 |
| 26 | C01G6.7 | *acs-7* | Fatty acid CoA synthetase family |  | 2.73 | 1.83 |
| 27 | Y34D9A.6 | *glrx-10* | Glutaredoxin |  | 2.46 | 1.82 |
| 28 | C45B11.3 | *dhs-18* | Dehydrogenases, short chain |  | 2.39 | 1.75 |
| 29 | C32H11.3 |  |  | Epoxide hydrolase 1, microsomal (xenobiotic) | 2.37 | 0.00 |
| 30 | C31C9.2 |  |  | Phosphoglycerate dehydrogenase | 2.27 | 0.74 |
| 31 | T04F8.7 |  |  |  | 2.21 | 1.27 |
| 32 | K11H12.3 |  |  |  | 2.19 | 0.00 |
| 33 | ZC443.6 | *ugt-16* | UDP-glucuronosyltransferase |  | 2.08 | 0.83 |
| 34 | R07B1.4 | *gst-36* | Glutathione S-transferase |  | 2.05 | 0.62 |
| 35 | B0024.4 |  |  | Involved in defense response | 2.04 | 2.16 |
| 36 | F58F9.7 |  |  | Acyl-CoA oxidase 3, pristanoyl | 2.04 | 1.19 |
| 37 | R107.7 | *gst-1* | Glutathione S-transferase |  | 1.88 | 1.77 |
| 38 | R12H7.3 | *skr-19* | Skp1 related (ubiquitin ligase complex component) |  | 1.85 | 0.90 |
| 39 | K08H10.1 | *lea-1* | Plant late embryo abundant (LEA) related |  | 1.81 | 1.05 |
| 40 | K08F4.7 | *gst-4* | Glutathione S-transferase |  | 1.81 | 1.64 |
| 41 | F27D9.2 |  |  |  | 1.76 | 0.00 |
| 42 | M01G12.12 | *rrf-2* | RNA-dependent RNA polymerase family |  | 1.75 | 2.74 |
| 43 | C33H5.13 |  |  |  | 1.62 | 1.49 |
| 44 | F55C12.7 | *tag-234* | Temporarily assigned gene name |  | 1.61 | 0.00 |
| 45 | T26C5.1 | *gst-13* | Glutathione S-transferase |  | 1.59 | 0.00 |
| 46 | F53E10.4 |  |  |  | 1.53 | 0.00 |
| 47 | C43C3.1 | *ifp-1* | Intermediate filament protein, class E |  | 1.46 | 0.00 |
| 48 | Y45G12C.2 | *gst-10* | Glutathione S-transferase |  | 1.42 | 0.88 |
| 49 | ZK1058.6 | *nit-1* | Nitrilase |  | 1.40 | 0.00 |
| 50 | F26E4.12 |  |  |  | 1.37 | 1.75 |
| 51 | T12B5.10 | *fbxa-60* | F-box A protein |  | 1.36 | 1.30 |
| 52 | F17A9.4 |  |  | FMN binding activity and oxidoreductase activity | 1.32 | 2.41 |
| 53 | F08G2.4 |  |  |  | 1.30 | 1.62 |
| 54 | K10C2.4 |  |  |  | 1.25 | -0.28 |
| 55 | C33G3.4 |  |  |  | 1.24 | 0.00 |
| 56 | F42G2.2 |  |  |  | 1.21 | 0.00 |
| 57 | F35E12.5 |  |  |  | 1.20 | 0.00 |
| 58 | F18G5.3 | *gpa-12* | G protein, alpha subunit |  | 1.17 | 0.00 |
| 59 | C35B1.5 |  |  |  | 1.17 | 0.00 |
| 60 | T10B10.8 |  |  |  | 1.15 | 0.00 |
| 61 | T13C5.6 |  |  | Phosphatidic acid phosphatase type 2 domain containing | 1.11 | 2.02 |
| 62 | M60.5 | *kqt-2* | Potassium channel, KvQLT family |  | 1.08 | 0.00 |
| 63 | K09H11.1 |  |  |  | 1.05 | 0.85 |
| 64 | Y116F11B.3 | *pcp-4* | Prolyl carboxypeptidase like |  | 1.03 | 0.00 |
| 65 | F11G11.2 | *gst-7* | Glutathione S-transferase |  | 1.00 | 1.00 |
| 66 | Y38F2AR.12 |  |  |  | 1.00 | 0.65 |
| 67 | C05E11.4 | *amt-1* | Ammonium transporter homolog |  | 0.82 | 0.00 |
| 68 | Y39C12A.1 |  |  |  | 0.81 | 0.00 |
| 69 | F10D2.9 | *fat-7* | Fatty acid desaturase |  | 0.77 | 0.39 |
| 70 | C54D1.4 | *alh-10* | Aldehyde dehydrogenase |  | 0.71 | 0.58 |
| 71 | W01A11.4 | *lec-10* | Galectin |  | 0.70 | 0.00 |
| 72 | F21F8.4 |  |  |  | 0.65 | 0.00 |
| 73 | K09E2.3 |  |  |  | 0.59 | 0.94 |
| 74 | F22H10.3 |  |  |  | 0.58 | 0.00 |
| 75 | K11D2.2 | *asah-1* | Acylsphingosine amidohydrolase |  | 0.58 | 0.00 |
| 76 | C54G6.5 | *spp-17* | Saposin-like protein family |  | 0.50 | 0.77 |
| 77 | C04F12.1 |  |  |  | 0.47 | 0.65 |
| 78 | K05C4.2 |  |  |  | 0.40 | 0.62 |
| 79 | ZK666.6 | *clec-60* | C-type lectin |  | 0.00 | 3.08 |
| 80 | C17H1.7 |  |  |  | 0.00 | 2.00 |
| 81 | F37B1.2 | *gst-12* | Glutathione S-transferase |  | 0.00 | 1.96 |
| 82 | C28G1.1 | *ubc-23* | Ubiquitin conjugating enzyme |  | 0.00 | 1.84 |
| 83 | Y1H11.2 | *gst-35* | Glutathione S-transferase |  | 0.00 | 1.84 |
| 84 | Y39G10AR.6 | *ugt-31* | UDP-glucuronosyltransferase |  | 0.00 | 1.40 |
| 85 | F42G2.4 | *fbxa-182* | F-box A protein |  | 0.00 | 1.21 |
| 86 | C06E1.1 |  |  |  | 0.00 | 1.17 |
| 87 | W03D2.6 |  |  |  | 0.00 | 1.10 |
| 88 | F55A4.8 |  |  |  | 0.00 | 1.02 |
| 89 | Y60A3A.18 | *skr-4* | Skp1 related (ubiquitin ligase complex component) |  | 0.00 | 0.93 |
| 90 | F43G9.9 | *cpn-1* | Calponin |  | 0.00 | 0.77 |
| 91 | C06H2.1 | *atp-5* | ATP synthase subunitATP synthase subunit |  | 0.00 | 0.13 |
| 92 | C53D5.5 |  |  |  | 0.00 | -0.51 |
| 93 | ZK1127.10 |  |  |  | 0.00 | -0.55 |
| 94 | F23B2.11 | *pcp-3* | Prolyl carboxypeptidase like |  | 0.00 | -0.72 |
| 95 | F40F9.9 | *aqp-4* | Aquaporin or aquaglyceroporin related |  | 0.00 | -0.86 |
| 96 | Y102A11A.3 |  |  |  | 0.00 | -0.98 |
| 97 | C05D9.2 | *lmp-2* | Intramembrane protease (IMPAS) family |  | 0.00 | -1.04 |
| 98 | C16H3.2 | *lec-9* | Galectin |  | 0.00 | -1.17 |
| 99 | Y53G8B.1 |  |  |  | 0.00 | -1.19 |
| 100 | K10H10.2 |  |  |  | 0.00 | -1.21 |
| 101 | H03A11.2 |  |  |  | 0.00 | -1.54 |
| 102 | M02F4.7 |  |  |  | 0.00 | -1.60 |
| 103 | F23B2.12 | *pcp-2* | Prolyl carboxypeptidase like |  | 0.00 | -2.45 |
| 104 | C30F8.2 | *vha-16* | Vacuolar H ATPase |  | -0.21 | 0.00 |
| 105 | ZK154.5 |  |  |  | -0.29 | 0.00 |
| 106 | R10E11.2 | *vha-2* | Vacuolar H ATPase |  | -0.31 | -0.31 |
| 107 | Y105C5B.15 |  |  |  | -0.35 | 0.00 |
| 108 | F09B9.3 | *erd-2* | ERD (yeast endoplasmic reticulum retention defective) related |  | -0.44 | 0.00 |
| 109 | ZC155.3 | *morc-1* | MORC (mouse microrchidia) family CW-type zinc finger protein |  | -0.46 | -0.21 |
| 110 | ZK455.1 | *aco-1* | Aconitase |  | -0.52 | -0.26 |
| 111 | C17H12.14 | *vha-8* | Vacuolar H ATPase |  | -0.53 | -0.54 |
| 112 | K12H4.7 |  |  |  | -0.55 | 0.00 |
| 113 | VW02B12L.1 | *vha-6* | Vacuolar H ATPase |  | -0.56 | -0.51 |
| 114 | K10C2.3 |  |  |  | -0.59 | 0.36 |
| 115 | K09A9.5 | *gas-1* | General anaesthetic sensitivity abnormal |  | -0.62 | -0.29 |
| 116 | Y37A1B.5 |  |  |  | -0.65 | 0.00 |
| 117 | F56F10.1 |  |  |  | -0.75 | -0.36 |
| 118 | F09B12.3 |  |  |  | -0.76 | -0.32 |
| 119 | Y119D3B.21 |  |  |  | -0.77 | -0.69 |
| 120 | K08F8.4 | *pah-1* | Phenylalanine hydroxylase |  | -0.79 | -0.30 |
| 121 | T04G9.5 |  |  |  | -0.83 | -0.56 |
| 122 | F20G2.2 |  |  |  | -0.85 | -0.45 |
| 123 | M28.8 |  |  |  | -0.87 | -1.09 |
| 124 | C49C3.4 |  |  |  | -0.88 | -0.92 |
| 125 | C14H10.1 |  |  |  | -0.90 | -0.86 |
| 126 | B0218.6 | *clec-51* | C-type lectin |  | -0.90 | 0.00 |
| 127 | C26B9.5 |  |  |  | -0.94 | -0.84 |
| 128 | F54F11.2 |  |  |  | -0.95 | -0.72 |
| 129 | T08H10.1 |  |  |  | -0.95 | -0.50 |
| 130 | F25E2.4 | *ifd-2* | Intermediate filament, D |  | -1.02 | 0.00 |
| 131 | T21C12.2 | *hpd-1* | 4-hydroxyphenylpyruvate dioxygenase |  | -1.04 | -0.81 |
| 132 | D1053.1 | *gst-42* | Glutathione S-transferase |  | -1.06 | -0.92 |
| 133 | W07A12.7 | *rhy-1* | Regulator of hypoxia-inducible factor (hif-1) |  | -1.16 | -1.14 |
| 134 | Y46H3B.1 |  |  |  | -1.21 | 0.00 |
| 135 | F32A5.3 |  |  |  | -1.24 | -0.93 |
| 136 | C18C4.3 | *ugt-48* | UDP-glucuronosyltransferase |  | -1.27 | 0.00 |
| 137 | F17A9.5 |  |  |  | -1.32 | 0.62 |
| 138 | T25C8.2 | *act-5* | Actin |  | -1.33 | -0.95 |
| 139 | B0310.5 | *ugt-46* | UDP-glucuronosyltransferase |  | -1.38 | -0.82 |
| 140 | F55E10.6 |  |  |  | -1.42 | -0.65 |
| 141 | K10B2.2 |  |  |  | -1.51 | 0.00 |
| 142 | D1086.3 |  |  |  | -1.54 | -1.23 |
| 143 | F13B6.2 |  |  |  | -1.58 | 0.00 |
| 144 | T25B6.2 |  |  |  | -1.58 | -0.74 |
| 145 | C12C8.2 |  |  |  | -1.65 | 0.00 |
| 146 | Y40D12A.2 |  |  |  | -1.69 | -1.65 |
| 147 | K10C2.1 |  |  |  | -1.86 | -1.62 |
| 148 | F07A5.3 |  |  |  | -1.87 | -1.05 |
| 149 | Y46H3B.2 |  |  |  | -2.04 | 0.00 |
| 150 | C55F2.1 |  |  |  | -2.05 | -1.03 |
| 151 | R03G5.5 |  |  |  | -2.10 | 0.00 |
| 152 | T15B7.16 |  |  |  | -2.11 | 0.00 |
| 153 | T16G12.1 |  |  |  | -2.40 | -2.22 |
| 154 | F58G6.7 |  |  |  | -2.64 | -1.05 |
| 155 | Y16B4A.2 |  |  |  | -2.85 | -2.23 |
| 156 | C09B8.4 |  |  |  | -2.88 | -1.45 |
| 157 | C08F11.8 | *ugt-22* | UDP-glucuronosyltransferase |  | -3.09 | -1.46 |
| 158 | F38B6.4 |  |  |  | -3.42 | -1.69 |
| 159 | F58G6.3 |  |  |  | -3.42 | -2.06 |
| 160 | K09C4.5 |  |  |  | -3.67 | -1.58 |

**(Electronic supplementary) Table A3.** DAF-16 target genes, with columns showing (from left to right) gene number, gene name, gene symbol, symbol explanation, additional information, log_2_-fold changes in expression (WT_Cd_ vs. WT_ctrl_ and *pmk-1*∆_Cd_ vs. *pmk-1*∆_ctrl_), and assignment to class I (1) or II (2) of DAF-16 target genes (Murphy et al. 2003). Gray-shaded cells indicate more positive or less negative Cd-induced expression changes in *pmk-1*∆ than in WT.

| **Number** | **Gene name** | **Gene symbol** | **Symbol explanation** | **Additional information** | **WT_Cd_ vs. WT_ctrl_** | ***pmk-1*∆_Cd_ vs. *pmk-1*∆_ctrl_** | **Gene class** |
| --- | --- | --- | --- | --- | --- | --- | --- |
| 1 | K10D11.2 |  |  | Epoxide hydrolase 1, microsomal (xenobiotic) | 12.09 | 0 | 2 |
| 2 | Y40B10A.6 | *comt-4* | Catechol-O-methyltransferase family |  | 11.89 | 0 | 1 |
| 3 | F47B8.4 |  |  | Glutaredoxin 5; involved in innate immune response and lipid storage | 11.28 | 10.92 | 1 |
| 4 | K11G9.6 | *mtl-1* | Metallothionein |  | 11.13 | 4.67 | 1 |
| 5 | C50F7.5 |  |  |  | 8.64 | 4.79 | 1 |
| 6 | C08E3.6 | *fbxa-163* | F-box A protein |  | 8.39 | 9.4 | 1 |
| 7 | Y46H3A.3 | *hsp-16.2* | Heat shock protein |  | 8.28 | 6.81 | 1 |
| 8 | K01A2.2 | *far-7* | Fatty acid/retinol binding protein |  | 6.14 | 4.42 | 1 |
| 9 | C45G7.3 | *ilys-3* | Invertebrate lysozyme |  | 5.95 | 5.75 | 1 |
| 10 | F53C3.5 |  |  |  | 5.55 | 2.84 | 1 |
| 11 | T24E12.5 |  |  |  | 5.43 | 0 | 1 |
| 12 | F35E12.7 | *dct-17* | DAF-16/FOXO controlled, germline tumor affecting |  | 5.25 | 0 | 2 |
| 13 | F28D1.3 | *thn-1* | Thaumatin family |  | 5.06 | 4.52 | 1 |
| 14 | F45D3.4 |  |  |  | 4.77 | 3.02 | 1 |
| 15 | F15E6.8 | *dct-7* | DAF-16/FOXO controlled, germline tumor affecting |  | 4.69 | 2.08 | 1 |
| 16 | AC3.7 | *ugt-1* | UDP-glucuronosyltransferase |  | 4.61 | 4.12 | 1 |
| 17 | C34C6.7 |  |  |  | 4.37 | 3.47 | 1 |
| 18 | ZK896.5 |  |  | Epoxide hydrolase 1, microsomal (xenobiotic) | 4.36 | 1.22 | 2 |
| 19 | C15H9.1 | *nnt-1* | Nicotinamide nucleotide transhydrogenase |  | 4.24 | 1.85 | 1 |
| 20 | F52H3.5 |  |  | Tetratricopeptide repeat domain 36 | 4.21 | 0 | 1 |
| 21 | C24B9.9 | *dod-3* | Downstream of DAF-16 (regulated by DAF-16) |  | 4.13 | 1.46 | 1 |
| 22 | C08E8.4 |  |  | Involved in innate immune response | 4.1 | 4.64 | 1 |
| 23 | T10B9.1 | *cyp-13A4* | Cytochrome P450 family |  | 3.9 | 2.57 | 1 |
| 24 | F47H4.10 | *skr-5* | Skp1 related (ubiquitin ligase complex component) |  | 3.76 | 3.36 | 1 |
| 25 | F09F7.6 |  |  |  | 3.62 | 0 | 1 |
| 26 | C46F4.2 | *acs-17* | Fatty acid CoA synthetase family |  | 3.6 | 1.61 | 1 |
| 27 | F53A9.1 |  |  |  | 3.58 | 1.55 | 1 |
| 28 | Y51A2D.11 | *ttr-26* | Transthyretin-related family domain |  | 3.4 | 2.02 | 1 |
| 29 | F15B9.1 | *far-3* | Fatty acid/retinol binding protein |  | 3.36 | 0 | 1 |
| 30 | C18A11.1 |  |  |  | 3.31 | 2.85 | 1 |
| 31 | C17H12.8 |  |  |  | 3.31 | 0 | 2 |
| 32 | F54F3.1 | *nid-1* | Nidogen (basement membrane protein) |  | 3.19 | 0 | 2 |
| 33 | ZK1251.2 | *ins-7* | Insulin related |  | 3.07 | 2.23 | 2 |
| 34 | F08B1.1 | *vhp-1* | VH1 dual-specificity phosphatase family |  | 3.03 | 1.14 | 1 |
| 35 | F56D6.2 | *clec-67* | C-type lectin |  | 2.96 | 0 | 2 |
| 36 | T21C9.8 | *ttr-23* | Transthyretin-related family domain |  | 2.92 | 0 | 1 |
| 37 | T19B10.2 |  |  |  | 2.8 | 0.85 | 1 |
| 38 | Y43C5A.3 |  |  |  | 2.77 | 2.52 | 1 |
| 39 | Y54G11A.6 | *ctl-1* | Catalase |  | 2.76 | 2.72 | 1 |
| 40 | C31A11.5 | *oac-6* | O-Acyltransferase homolog |  | 2.69 | 0 | 2 |
| 41 | T19D12.4 |  |  | Involved in innate immune response | 2.67 | 0 | 2 |
| 42 | Y54G11A.5 | *ctl-2* | Catalase |  | 2.66 | 1.29 | 1 |
| 43 | F13D11.4 |  |  | 3-Beta-hydroxy-delta 5-steroid dehydrogenase | 2.65 | 1.34 | 1 |
| 44 | Y40B10A.2 |  |  | *comt-3*, catechol-O-methyltransferase domain containing | 2.57 | 3.02 | 1 |
| 45 | T10E10.2 | *col-167* | Collagen |  | 2.48 | 0 | 2 |
| 46 | C25D7.5 |  |  | Involved in innate immune response | 2.44 | 2.35 | 2 |
| 47 | W05H9.1 |  |  |  | 2.44 | 1.26 | 1 |
| 48 | F38E11.2 | *hsp-12.6* | Heat shock protein |  | 2.42 | 0 | 1 |
| 49 | T10H4.11 | *cyp-34A2* | Cytochrome P450 family |  | 2.4 | 1.48 | 1 |
| 50 | C45B11.3 | *dhs-18* | Dehydrogenases, short chain |  | 2.39 | 1.75 | 1 |
| 51 | C30G7.1 | *hil-1* | Histone H1 Like |  | 2.38 | 1.71 | 1 |
| 52 | C05E4.9C05E4.9 | *gei-7*, *icl-1* | Isocitrate lyase homolog |  | 2.36 | 1.38 | 1 |
| 53 | M163.3 | *his-24* | Histone |  | 2.31 | 1.07 | 2 |
| 54 | F13H6.3 |  |  | Carboxylesterase 2 | 2.29 | 1.7 | 2 |
| 55 | F28D1.5 | *thn-2* | Thaumatin family |  | 2.25 | 1.49 | 1 |
| 56 | C25E10.8 |  |  | Secreted TIL-domain protease inhibitor | 2.22 | 0.78 | 1 |
| 57 | ZK550.6 |  |  | Phytanoyl-CoA hydroxylase | 2.13 | 0.93 | 1 |
| 58 | T28F4.5 |  |  | Death associated protein 1 | 2.09 | 1.48 | 1 |
| 59 | C34D10.2 |  |  | Unkempt family zinc finger-like | 2.02 | 1.47 | 1 |
| 60 | C37H5.2 | *abhd-5.1* | Abhydrolase domain containing homolog |  | 1.99 | 2.46 | 1 |
| 61 | W01B11.6 |  |  |  | 1.97 | 0 | 1 |
| 62 | F52H3.7 | *lec-2* | Galectin |  | 1.95 | 1.52 | 1 |
| 63 | K08D8.5 |  |  |  | 1.91 | 0 | 2 |
| 64 | B0365.6 | *clec-41* | C-type lectin |  | 1.91 | 0 | 2 |
| 65 | JC8.8 | *ttr-51* | Transthyretin-related family domain |  | 1.89 | 1.65 | 1 |
| 66 | C49C3.9 |  |  |  | 1.85 | 0.91 | 2 |
| 67 | K08F4.7 | *gst-4* | Glutathione S-Transferase |  | 1.81 | 1.64 | 1 |
| 68 | F10D2.11 | *ugt-41* | UDP-glucuronosyltransferase |  | 1.79 | 0 | 1 |
| 69 | F46G10.3 | *sir-2.3* | Yeast SIR related |  | 1.77 | 0 | 2 |
| 70 | F36F2.2 |  |  |  | 1.74 | 1.39 | 1 |
| 71 | F53C11.1 |  |  |  | 1.72 | 0 | 2 |
| 72 | R03E9.1 | *mdl-1* | MAD-like |  | 1.67 | 0 | 1 |
| 73 | T05D4.2 |  |  |  | 1.67 | 0 | 2 |
| 74 | F19H8.1 | *tps-2* | Trehalose 6-phosphate synthase |  | 1.63 | 1.16 | 1 |
| 75 | K08D8.3 |  |  |  | 1.63 | 0.71 | 2 |
| 76 | C17G10.5 | *lys-8* | Lysozyme |  | 1.63 | 0 | 1 |
| 77 | Y43F8A.3 |  |  | *nceh-1*, neutral cholesterol ester hydrolase homolog | 1.59 | 2.03 | 1 |
| 78 | C44H9.5 |  |  |  | 1.59 | 1.19 | 1 |
| 79 | C52D10.9 | *skr-8* | Skp1 related (ubiquitin ligase complex component) |  | 1.59 | 0 | 2 |
| 80 | Y56A3A.33 |  |  |  | 1.58 | 1.22 | 1 |
| 81 | F08B12.4 |  |  |  | 1.55 | 1.07 | 1 |
| 82 | C10C5.4 |  |  | Aminoacylase | 1.54 | 2 | 2 |
| 83 | C40H1.5 | *ttr-5* | Transthyretin-related family domain |  | 1.51 | 1.02 | 1 |
| 84 | M01H9.3 |  |  |  | 1.49 | 0 | 1 |
| 85 | M01H9.3 |  |  |  | 1.4 | 0 | 1 |
| 86 | C53B7.3 |  |  |  | 1.39 | 1.27 | 1 |
| 87 | B0244.2 | *ida-1* | Related to islet cell diabetes autoantigen |  | 1.38 | 0.95 | 1 |
| 88 | F53C3.12 | *bcmo-2* | Beta-carotene 15,15'- monooxygenase |  | 1.36 | 0 | 1 |
| 89 | R13H4.5 |  |  |  | 1.32 | 1.32 | 1 |
| 90 | T23B3.2 |  |  |  | 1.29 | 2.22 | 1 |
| 91 | T13F2.8 | *cav-1* | CaveolinCAVeolin |  | 1.26 | 1.07 | 2 |
| 92 | T21D12.9 | *sma-10* | SMAll |  | 1.23 | 0.96 | 1 |
| 93 | F35E12.5 |  |  |  | 1.2 | 0 | 2 |
| 94 | B0218.8 | *clec-52* | C-type lectin |  | 1.18 | 0.71 | 1 |
| 95 | T23H2.2 | *snt-4* | Synaptotagmin |  | 1.18 | 0.7 | 1 |
| 96 | C05C12.4 |  |  |  | 1.17 | 0 | 1 |
| 97 | F09F7.7 |  |  |  | 1.12 | 1.29 | 1 |
| 98 | K01A2.5 |  |  |  | 1.12 | 1.26 | 2 |
| 99 | K08D8.6 |  |  |  | 1.11 | 0.36 | 2 |
| 100 | Y41D4B.16 |  |  | *hpo-6*, hypersensitive to pore-forming toxin | 1.11 | 0 | 2 |
| 101 | F52E4.5 |  |  |  | 1.06 | 3.47 | 1 |
| 102 | Y71G12B.4 | *pghm-1* | Peptidylglycine-alpha-hydroxylating monooxygenase |  | 1.04 | 0.82 | 1 |
| 103 | C35E7.5 |  |  |  | 1.03 | 0 | 2 |
| 104 | F58B3.9 | *ttr-50* | Transthyretin-related family domain |  | 1.02 | 1.03 | 2 |
| 105 | M02D8.4 | *asns-2* | Asparagine synthetase |  | 0.97 | 0 | 1 |
| 106 | W08D2.4 | *fat-3* | Fatty acid desaturase |  | 0.95 | 0.75 | 1 |
| 107 | M04G12.2 | *cpz-2* | Cathepsin Z |  | 0.89 | 0.79 | 1 |
| 108 | T21D12.9 | *sma-10* | SMAll |  | 0.87 | 0 | 1 |
| 109 | F21C10.10 |  |  |  | 0.86 | 0.7 | 1 |
| 110 | F35E12.10 |  |  |  | 0.83 | 0.59 | 2 |
| 111 | F15E6.4 |  |  |  | 0.79 | 0 | 1 |
| 112 | F10D2.9 | *fat-7* | Fatty acid desaturase |  | 0.77 | 0.39 | 1 |
| 113 | C09F12.1 | *clc-1* | Claudin-like in Caenorhabditis |  | 0.77 | 0 | 2 |
| 114 | W02B12.1 |  |  |  | 0.77 | 0 | 2 |
| 115 | F54E2.1 |  |  |  | 0.76 | 0.87 | 2 |
| 116 | F54F7.3 |  |  |  | 0.73 | 1.29 | 1 |
| 117 | T18D3.4 | *myo-2* | Myosin heavy chain structural genes |  | 0.73 | 0.37 | 2 |
| 118 | W01A11.4 | *lec-10* | Galectin |  | 0.7 | 0 | 2 |
| 119 | W05E10.4 | *tre-3* | Trehalase |  | 0.63 | 0 | 2 |
| 120 | C05D9.1 | *snx-1* | Sorting nexin |  | 0.58 | 0.71 | 1 |
| 121 | F54D5.3 |  |  |  | 0.58 | 0.27 | 1 |
| 122 | K11D2.2 | *asah-1* | Acylsphingosine amidohydrolase |  | 0.58 | 0 | 1 |
| 123 | W02D3.1 | *cytb-5.2* | Cytochrome B |  | 0.57 | 0.5 | 1 |
| 124 | C32E8.11 | *ubr-1* | UBR E3 ubiquitin ligase homolog |  | 0.56 | 0.39 | 2 |
| 125 | ZK1320.2 |  |  |  | 0.55 | 0 | 1 |
| 126 | F49E12.2 | *dod-23* | Downstream of DAF-16 (regulated by DAF-16) |  | 0.51 | 0 | 2 |
| 127 | C52E4.1 | *cpr-1* | Cysteine protease related |  | 0.45 | -0.16 | 1 |
| 128 | C02A12.4 | *lys-7* | Lysozyme |  | 0.44 | 0.37 | 1 |
| 129 | H22K11.1 | *asp-3* | Aspartyl protease |  | 0.43 | 0.25 | 1 |
| 130 | W06D12.3 | *fat-5* | Fatty acid desaturase |  | 0.42 | 0 | 1 |
| 131 | T13B5.3 | *pho-14* | Intestinal acid phosphatase |  | 0.37 | 0.44 | 1 |
| 132 | Y116A8C.35 | *uaf-2* | U2AF splicing factor |  | 0.33 | 0.27 | 2 |
| 133 | Y49E10.1 | *rpt-6* | Proteasome regulatory particle, ATPase-like |  | 0.29 | 0.46 | 2 |
| 134 | Y32H12A.8 |  |  |  | 0.26 | -0.4 | 1 |
| 135 | T04H1.2 |  |  |  | 0.24 | 0.49 | 1 |
| 136 | C08F11.11 |  |  |  | 0.24 | 0.18 | 1 |
| 137 | Y54E10A.2 | *cogc-1* | Conserved oligomeric Golgi (COG) component |  | 0.23 | 0 | 2 |
| 138 | D2045.6 | *cul-1* | Cullin |  | 0.2 | 0.31 | 2 |
| 139 | R05F9.10 | *sgt-1* | Small glutamine-rich tetratrico repeat protein |  | 0.16 | 0 | 2 |
| 140 | C42D8.2 | *vit-2* | Vitellogenin structural genes (yolk protein genes) |  | 0.16 | -0.14 | 2 |
| 141 | ZK355.3 |  |  |  | 0 | 11.54 | 1 |
| 142 | C08E3.4 | *fbxa-161* | Pseudogene (F-box A protein ) |  | 0 | 11.27 | 1 |
| 143 | F26D11.2 |  |  |  | 0 | 3.77 | 1 |
| 144 | PDB1.1 |  |  |  | 0 | 1.37 | 1 |
| 145 | F21F3.3 | *icmt-1* | Isoprenylcysteine carboxyl methyltransferase related |  | 0 | 1.23 | 1 |
| 146 | C10C5.5 |  |  |  | 0 | 1.14 | 2 |
| 147 | W03D2.6 |  |  |  | 0 | 1.1 | 1 |
| 148 | F46H5.7 |  |  |  | 0 | 0.98 | 1 |
| 149 | C25E10.9 | *swm-1* | Sperm activation without mating |  | 0 | 0.94 | 1 |
| 150 | R107.8 | *lin-12* | Abnormal cell lineage |  | 0 | 0.89 | 2 |
| 151 | ZC247.1 |  |  |  | 0 | 0.73 | 1 |
| 152 | R05F9.13 | *msp-31* | Major sperm protein |  | 0 | 0.72 | 1 |
| 153 | F53F4.13 |  |  |  | 0 | 0.56 | 1 |
| 154 | T16G12.4 |  |  |  | 0 | 0.54 | 2 |
| 155 | F57C2.4 |  |  |  | 0 | 0.46 | 2 |
| 156 | K08B4.6 | *cpi-1* | Cysteine protease inhibitor |  | 0 | 0.44 | 1 |
| 157 | T23G7.3 |  |  |  | 0 | 0.28 | 1 |
| 158 | Y45F10A.2 | *puf-3* | PUF (Pumilio/FBF) domain-containing |  | 0 | -0.22 | 2 |
| 159 | VC5.3 | *npa-1* | Nematode polyprotein allergen related |  | 0 | -0.45 | 1 |
| 160 | ZK1127.10 | *cth-2* | Cystathionine gamma lyase |  | 0 | -0.55 | 2 |
| 161 | C25H3.11 |  |  |  | 0 | -0.85 | 2 |
| 162 |  |  |  |  | 0 | -0.92 | 1 |
| 163 | ZK6.10 | *dod-19* | Downstream of DAF-16 (regulated by DAF-16) |  | 0 | -0.94 | 2 |
| 164 | T02B5.1 |  |  |  | 0 | -1.12 | 1 |
| 165 | Y53G8B.1 |  |  |  | 0 | -1.19 | 1 |
| 166 | F15E11.12 | *pud-4* | Protein up-regulated in Daf-2(gf) |  | 0 | -1.44 | 2 |
| 167 | M02F4.7 | *clec-265* | C-type lectin |  | 0 | -1.6 | 2 |
| 168 | ZK6.11 |  |  |  | 0 | -2.09 | 2 |
| 169 | F23B2.12 | *pcp-2* | Prolyl carboxypeptidase like |  | 0 | -2.45 | 2 |
| 170 | ZK384.2 | *scl-20* | SCP-like extracellular protein |  | 0 | -2.94 | 1 |
| 171 | C04F6.1 | *vit-5* | Vitellogenin structural genes (yolk protein genes) |  | -0.05 | -0.45 | 2 |
| 172 | H19N07.1 | *erfa-3* | Eukaryotic release factor homolog |  | -0.13 | 0 | 2 |
| 173 | D1054.11 |  |  |  | -0.15 | 0.19 | 2 |
| 174 | T03E6.7 | *cpl-1* | Cathepsin L family |  | -0.16 | 0.14 | 2 |
| 175 | C07H6.5 | *cgh-1* | Conserved germline helicase |  | -0.22 | -0.35 | 2 |
| 176 | F52E1.1 | *pos-1* | Posterior segregation |  | -0.23 | 0 | 2 |
| 177 | F18A1.7 |  |  |  | -0.23 | 0 | 2 |
| 178 | H12C20.2 | *pms-2* | PMS (post meiotic segregation) family |  | -0.26 | -0.25 | 2 |
| 179 | F57F5.1 |  |  |  | -0.26 | -0.55 | 2 |
| 180 | F49E12.1 | *skpo-1* | ShK domain and peroxidase domain containing protein |  | -0.3 | 0 | 2 |
| 181 | T10B11.3 | *ztf-4* | Zinc finger putative transcription factor family |  | -0.33 | 0 | 2 |
| 182 | F34D10.4 |  |  |  | -0.34 | 0 | 2 |
| 183 | Y62H9A.3 |  |  |  | -0.37 | 0 | 2 |
| 184 | Y14H12B.2 |  |  |  | -0.37 | 0 | 2 |
| 185 | C39D10.7 |  |  |  | -0.39 | -0.25 | 2 |
| 186 | K06B9.4 |  |  |  | -0.46 | 0 | 2 |
| 187 | K01C8.5 | *gei-14* | GEX interacting protein |  | -0.52 | 0 | 2 |
| 188 | C54D10.3 |  |  |  | -0.53 | 0 | 1 |
| 189 | ZC416.6 |  |  |  | -0.53 | -0.5 | 2 |
| 190 | K12H4.7 |  |  |  | -0.55 | 0 | 2 |
| 191 | F59F4.4 | *acl-1* | Acyltransferase-like |  | -0.55 | -0.53 | 1 |
| 192 | VW02B12L.1 | *vha-6* | Vacuolar H ATPase |  | -0.56 | -0.51 | 2 |
| 193 | M03F4.7 | *calu-1* | Calumenin (calcium-binding protein) homolog |  | -0.61 | -0.25 | 2 |
| 194 | C50E3.12 |  |  |  | -0.65 | 0 | 2 |
| 195 | W06D4.1 | *hgo-1* | Homogentisate oxidase |  | -0.66 | -0.45 | 1 |
| 196 | K12G11.3 | *sodh-1* | Sorbitol dehydrogenase family |  | -0.68 | -0.81 | 1 |
| 197 | F58G1.4 | *dct-18* | DAF-16/FOXO controlled, germline tumor affecting |  | -0.69 | 0 | 2 |
| 198 | C05D2.8 |  |  |  | -0.7 | 0 | 2 |
| 199 | C08B11.4 | *nrf-6* | Nose resistant to fluoxetine |  | -0.72 | -0.5 | 2 |
| 200 | T03F6.1 | *qdpr-1* | Quinoid dihydropteridine reductase |  | -0.73 | -0.34 | 2 |
| 201 | Y62H9A.4 |  |  |  | -0.73 | -0.56 | 2 |
| 202 | C25F6.3 | *dpyd-1* | Dihydropyrimidine dehydrogenase |  | -0.73 | -0.75 | 2 |
| 203 | T11F9.12 |  |  |  | -0.75 | 0.5 | 2 |
| 204 | F09B12.3 |  |  |  | -0.76 | -0.32 | 1 |
| 205 | F28H7.3 |  |  |  | -0.76 | -1.12 | 2 |
| 206 | F59D8.1 | *vit-3* | Vitellogenin structural genes (yolk protein genes) |  | -0.82 | -0.87 | 2 |
| 207 | C06B3.3 | *cyp-35C1* | Cytochrome P450 family |  | -0.83 | 0 | 2 |
| 208 | F59D8.2 | *vit-4* | Vitellogenin structural genes (yolk protein genes) |  | -0.87 | -0.91 | 2 |
| 209 | R09B5.6 | *hacd-1* | Hydroxy-acyl-CoA dehydrogenase |  | -0.88 | 0 | 1 |
| 210 | F54F11.2 | *nep-17* | Neprilysin metallopeptidase family |  | -0.95 | -0.72 | 2 |
| 211 | T01B11.2 |  |  |  | -0.99 | -0.51 | 1 |
| 212 | F18E3.7 | *ddo-2* | D-aspartate (D) oxidase |  | -1.01 | -0.69 | 1 |
| 213 | K10B3.8 | *gpd-2* | GPD (glyceraldehyde 3-phosphate dehydrogenase) |  | -1.02 | -0.55 | 1 |
| 214 | T21C12.2 | *hpd-1* | 4-Hydroxyphenylpyruvate dioxygenase (HPD) |  | -1.04 | -0.81 | 2 |
| 215 | ZK896.7 | *clec-186* | C-type lectin |  | -1.13 | -0.73 | 2 |
| 216 | C54D10.1 | *cdr-2* | Cadmium responsive |  | -1.17 | 0.89 | 1 |
| 217 | F28F8.2 | *acs-2* | Fatty acid CoA synthetase family |  | -1.18 | 0 | 1 |
| 218 | F17A9.5 |  |  |  | -1.32 | 0.62 | 1 |
| 219 | F38A1.5 | *clec-166* | C-type lectin |  | -1.39 | -1.08 | 1 |
| 220 | F28B4.3 |  |  |  | -1.4 | -1.24 | 2 |
| 221 | C03G6.15 | *cyp-35A2* | Cytochrome P450 family |  | -1.54 | 0 | 2 |
| 222 | D1086.3 |  |  |  | -1.54 | -1.23 | 1 |
| 223 | C12C8.2 | *cbl-1* | Cystathionine beta lyase |  | -1.65 | 0 | 2 |
| 224 | EGAP2.3 | *pho-1* | Intestinal acid phosphatase |  | -1.81 | -0.95 | 2 |
| 225 | C15H9.7 | *flu-2* | Abnormal fluorescence under UV illumination |  | -1.9 | -1.46 | 2 |
| 226 | T17H7.1 |  |  |  | -1.92 | -1.55 | 1 |
| 227 | T25C12.3 |  |  |  | -1.93 | -1.78 | 2 |
| 228 | W10G6.3 | *mua-6* | Muscle attachment abnormal |  | -2.28 | -1.15 | 1 |
| 229 | Y38H6C.1 | *dct-16* | DAF-16/FOXO controlled, germline tumor affecting |  | -2.4 | -1.48 | 2 |
| 230 | T16G12.1 |  |  |  | -2.4 | -2.22 | 2 |
| 231 | F28A12.4 | *asp-13* | Aspartyl protease |  | -2.61 | -1.72 | 1 |
| 232 | B0286.3 |  |  |  | -2.63 | -0.98 | 1 |
| 233 | K04E7.2 | *pept-1* | Peptide transporter family |  | -2.98 | -2.27 | 2 |
| 234 | C08F11.8 | *ugt-22* | UDP-glucuronosyltransferase |  | -3.09 | -1.46 | 2 |
| 235 | K09D9.2 | *cyp-35A3* | Cytochrome P450 family |  | -3.22 | 0 | 2 |
| 236 | F57F4.3 | *gfi-1* | GEI-4 (Four) interacting protein |  | -3.23 | -2.57 | 2 |
| 237 | F46E10.1 | *acs-1* | Fatty acid CoA synthetase family |  | -3.26 | -1.82 | 2 |
| 238 | F57F4.4 |  |  |  | -3.34 | -2.34 | 2 |
| 239 | F38B6.4 |  |  |  | -3.42 | -1.69 | 1 |
| 240 | K09C4.5 |  |  |  | -3.67 | -1.58 | 1 |
| 241 | K06A4.1 | *nas-3* | Nematode astacin protease |  | -12.51 | -5.19 | 2 |
